# Self-reported gastrointestinal adverse effects of non-steroidal anti-inflammatory drugs in female students with dysmenorrhoea at Makerere University: prevalence, discontinuation and associated factors. a cross sectional study

**DOI:** 10.1136/bmjopen-2023-079660

**Published:** 2024-06-06

**Authors:** Solomon Gobba, Winnie Kibone, Ronald Kiguba

**Affiliations:** 1 Makerere University, Kampala, Uganda; 2 Pharmacology and Therapeutics, Makerere University College of Health Sciences, Kampala, Uganda

**Keywords:** Dysmenorrhea, Adverse events, REPRODUCTIVE MEDICINE

## Abstract

**Background:**

Primary dysmenorrhoea occurs in up to 50% of menstruating females. Non-steroidal anti-inflammatory drugs (NSAIDs) are the most commonly used therapeutic remedies for dysmenorrhoea in Uganda. However, NSAIDs are associated with a 3–5 fold increase in the risk of gastrointestinal (GI) adverse drug effects.

**Objectives:**

We aimed to determine the prevalence and associated factors of self-reported NSAID-related GI adverse effects in female students who use NSAIDs in managing dysmenorrhoea-associated pain at Makerere University.

**Design:**

A cross-sectional study.

**Setting:**

Makerere University’s main campus, situated North of Kampala, Uganda.

**Participants:**

314 female students pursuing an undergraduate programme at Makerere University and residing in different halls of residence and hostels.

**Outcomes:**

Social demographic data, menstrual history and treatment data.

**Results:**

Overall, 314 valid responses were received from female students with a median age of 22 years (IQR: 18–29 years). The median age at menarche was 13 years (IQR: 9–18 years). 41% (n=129/314) of the respondents had used medication for dysmenorrhoea and 32% (n=41/129) of whom reported NSAID-associated GI adverse effects with nausea being the most frequently reported (44%, n=18/41)

Factors independently associated with GI adverse effects were: age at menarche (p=0.026), duration of menstruation (p=0.030) and use of ibuprofen (p=0.005). Females taking ibuprofen for dysmenorrhoea were about four times as likely to have NSAID-associated GI adverse effects (adjusted OR 3.87, 95% CI 1.51 to 9.91) than those who did not receive ibuprofen. Logistic regression was used to determine factors associated with self-reported adverse effects of NSAIDs among the female students. A p<0.05 was considered statistically significant.

**Conclusion:**

We found a considerably high prevalence of NSAID-related GI adverse effects driven by factors such as age at menarche and ibuprofen use.

**STRENGTHS AND LIMITATIONS OF THIS STUDY**

- The study focuses on a public health issue (non-steroidal anti-inflammatory drugs (NSAIDS)-related gastrointestinal (GI) effects) in a specific population (female students with dysmenorrhoea) in Uganda, where this information is limited.
- The study has clear objectives, elaborate study design and a well-defined methodology to determine the prevalence and associated factors of NSAID-related GI effects.
- The online survey method excludes students without internet access, potentially leading to a biased sample that may not represent the entire student population.
- Relying on self-reported data on medication use and GI effects can introduce some bias due to potential recall errors or misinterpretations.

## Introduction

Dysmenorrhoea is defined as painful menstrual cramps and is classified into primary dysmenorrhoea without any evident pathology and secondary dysmenorrhoea with a pathology.^1 2^ Pinpointing the exact prevalence remains elusive, as studies conducted in diverse populations show varying rates, spanning from 20% to 94%^3^ Primary dysmenorrhoea occurs in up to 50% of menstruating females and causes significant disruption in quality of life and absenteeism from school and workplaces affecting their productivity.^4^ The pain is attributed to uterine prostaglandins released by endometrial cells during uterine wall sloughing before the onset of menstruation.^5^ On account of the high prevalence of dysmenorrhoea with a global prevalence ranging between 20% and 90%, dysmenorrhoea is a serious public health burden.^6^ Studies have documented an equally higher prevalence of dysmenorrhoea estimated at 65.4%, 72.7% and 83.6% in Egypt, Ghana and Turkey, respectively.^7–9^ In Uganda, the prevalence of dysmenorrhoea has been estimated at 75.8%.^10^

Non-steroidal anti-inflammatory drugs (NSAIDs) remain the most commonly used therapeutic remedies for dysmenorrhoea in Uganda because of their affordability, convenience of use and efficacy.^11^ NSAIDs are commonly used as over-the-counter drugs and may often not be prescribed.^12 13^ NSAIDs relieve symptoms in up to 70% of women when used correctly. Furthermore, NSAIDs are the recommended first-line treatment in management of dysmenorrhoea unless there are contraindications such as history of hypersensitivity to aspirin or other NSAIDs, serious comorbidity and gastrointestinal (GI) ulcers or bleeding.^5^

The utilisation of NSAIDs has been linked to a significant 3–5 fold increase in the risk of GI adverse effects.^14^ Dyspepsia with pyrosis, abdominal pain, nausea, anorexia, gastric erosions, ulcers, perforation, GI haemorrhage, which may result in anaemia, are some of the commonly observed GI adverse effects of NSAID therapy.^15^ Studies have explored GI complications of NSAID-related treatment in cases of rheumatoid arthritis and osteoarthritis in the elderly^16^ but little is known in relation to GI adverse drug effects related to NSAIDs therapy in the management of dysmenorrhoea. Furthermore, university students practice more self-medication using NSAIDs for dysmenorrhoea. In fact, a recent publication showed a high prevalence of self-medication estimated at 37% commonly mefenamic acid (58%).

This study aimed to determine the commonly used NSAIDs, the prevalence of self-reported NSAID-related GI adverse effects and the factors associated with self-reported NSAID-related GI adverse effects during the management of dysmenorrhoea among female students at Makerere University, Kampala, Uganda.

## Methods

### Study design

A descriptive cross-sectional study was conducted, between 14 October 2020 and 29 October 2020 to determine the prevalence and factors associated with GI adverse effects to NSAIDs usage in the management of dysmenorrhoea among female students at Makerere University.

### Study setting

The study was conducted at Makerere University’s main campus, situated North of Kampala, the capital city of Uganda, at the coordinates 0.3293° N, 32.5711° E. Makerere University is the largest public university in Uganda, attracting students from various regions of the country. The university accommodates over 40 000 students, both resident and non-resident. The main campus features twelve Halls of Residence, with six for male students and three for female students. Medical students are accommodated at the Mulago Hospital Complex. Approximately 5000 resident undergraduates and 100 graduate students, accounting for 13% of the university’s registered students, reside in the Halls of Residence. The remaining students either live in private hostels or commute from their homes. The age range of the majority of students falls between 20 and 30 years, encompassing a diverse representation of socioeconomic statuses.

### Study population

Female students pursuing an undergraduate programme at Makerere University and residing in different halls of residence and hostels. The sample size of 314 participants was calculated using the modified Kish and Leslie formula^17^ for finite population size, with an estimated prevalence of dysmenorrhoea among university students (75.8%), and a 10% adjustment was made to cater for non-response.

### Selection criteria

All registered female students of Makerere University aged 18 years and older were eligible for the study after informed consent. A purposive sampling technique was used to select students for the study. Female students with primary or secondary amenorrhoea, in menopause, with mental incapacitation or without access to internet were excluded from the study.

### Data collection

We used a self-administered questionnaire to collect data for this study. The questionnaire was pretested for comprehension and appropriateness among 15 students and adjusted accordingly to ensure quality data collection. The data were collected online via a Google survey document where participants received the survey information via a link shared with them in their WhatsApp groups and in their personal email inboxes. Eligible participants consented to the study and completed the survey. The submitted survey responses were automatically stored in an online data bank, accessible only to the principal investigator (PI) and the supervisor. Additionally, the data were backed up on the PI’s Google Drive to ensure data security. The questionnaire variables were constituted in three parts: sociodemographic information, menstrual history and treatment variables. These sections aimed to gather comprehensive data relevant to the study objectives.

### Study variables

Independent variables: social demographics (age, and history of pregnancy), menstrual history (dysmenorrhoea, menarche, regularity and duration of menstrual periods) and treatment (painkillers used and their source, side effects).

### Dependent variables: NSAID-related GI adverse effects

#### Data quality control

To ensure that high-quality data were captured from the volunteers, the data collection tool was pretested and adjusted to ensure convenience and ease of manoeuvre. Checks were incorporated within the document to ensure all questions were responded to and correctly answered to reduce the margins of error.

#### Data management

Data collection tools were checked for completeness and instantly submitted to a central data bank that tallied all responses received from the participants. The central databank was synchronised and backed up on the PI’s Google Drive for secondary storage. On completion of the survey, the data were translated into Excel for data cleaning and initial analysis. The data were then exported to STATA Software (StataCorp) for further analysis.

#### Data analysis

All analyses were performed using Microsoft Excel 2016 and STATA Software. Numerical data were summarised as means and SD if normally distributed; otherwise, median and IQRs were used for non-normally distributed data. Categorical data were summarised as frequencies and proportions with their 95% CIs. Associations between independent variables and dependent variables were assessed using the χ^2^ test and multivariable analysis using STATA V.15.1 software. A p<0.05 was considered statistically significant.

### Patient and public involvement

Patients and the public were at the centre of this research, our research questions and outcome measures were shaped by findings from previous studies which often reflect patient-reported outcomes, adverse events and medication-related errors. We aimed to develop research objectives that were meaningful, relevant and actionable in addressing medication safety issues from a patient-centred standpoint.

We conducted a questionnaire pretest session with a select group of participants to refine study questionnaire which ensured that the study design was sensitive to patient preferences and experiences.

The results of the study will be disseminated to study participants and the general public through various channels to ensure accessibility and comprehension. This will include the distribution of lay summaries written in plain language, presentations at patient-focused conferences and publication of study findings in open-access journals.

## Results

### Characteristics of participants

Overall, a total of 314 participants responded to the study. The median age was 22 years (IQR: 21–23). The majority came from nuclear families (62%, 194/314). Only 8% (26/314) of the participants had a history of pregnancy. The median age at menarche was 13 years (IQR: 12–14) with a range of 9–18 years and more than half (55%, 173/314) experienced regular periods every time. Nearly all female students were aware of dysmenorrhoea (99%, 310/314). Table 1 summarises the baseline characteristics of participants.

**Table 1:** Demographic and menstrual characteristics of participants

Variable	Frequency (n)	%
Age of respondents, years (median, IQR)	22	21–23
18–21	70	22.3
22–25	168	53.5
26–29	40	12.7
30 and above	36	11.5
Age at menarche, years (median, IQR)	13	12–14
9–11	24	7.6
12–14	206	65.6
15 and above	84	26.8
Type of family		
Nuclear	194	61.8
Extended	81	25.8
Single parent	37	11.8
Divorced parents	2	0.6
History of pregnancy		
Yes	26	8.3
No	288	91.7
Awareness on dysmenorrhoea		
Yes	310	98.7
No	4	1.3
Regular menstrual periods		
Every time	173	55.1
Most times	91	29
Sometimes	34	10.8
Rarely	16	5.1
Duration of menstrual periods (days)		
1–2	10	3.2
3–4	95	30.3
5–6	76	24.2
7–8	19	6.1
9 and above	1	0.3

### Patterns of dysmenorrhoea and medication use

**Figure 1:**
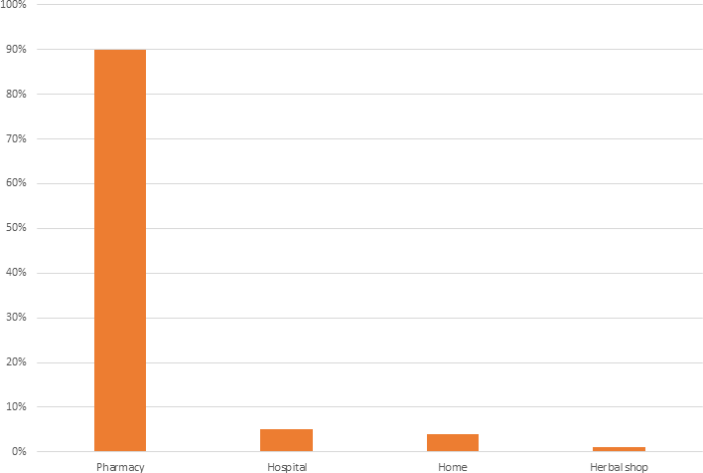
Sources of medication for menstrual cramps relief among female students at Makerere University.

The majority of participants (42%, 132/314) reported experiencing menstrual cramps every time and about a quarter (23%, 71/314) reported using medicines to reduce pain during menstrual pain every time. Most (87%, 273/314) of the participants reported experiencing menstrual cramps in the past 3 months of whom nearly half (47%, 129/273) reported using a medication for menstrual cramps with Ibuprofen and paracetamol being the most frequently used medications (40%, 52/129 and 40%, 52/129, respectively). The majority (90%, 116/129) bought the medication from pharmacies. Figure 1 shows the sources of the medication. Table 2 summarises the patterns of dysmenorrhoea and medication use among female students at Makerere University.

**Table 2:** Patterns of dysmenorrhoea and medication use among female students at Makerere University

Variable	Frequency	%
Experienced menstrual pains/cramps/dysmenorrhoea		
Every time	132	42.0
Most times	71	22.6
Sometimes	76	24.2
Rarely	35	11.2
Frequency of use of medicines (pain killers) to reduce pain during your menstrual period		
Every time	71	22.6
Most times	52	16.6
Sometimes	60	19.1
Rarely	131	41.7
Experienced menstrual cramps in the past 3 months		
Yes	273	86.9
No	41	13.1
How long ago the most recent period was (days)		
1–5	180	57.32
6–10	98	31.21
11 and above	36	11.47
Use of medication		
Yes	129	47.3
No	144	52.8
Medicines used for menstrual pains/cramps/dysmenorrhoea		
Ibuprofen	52	40.3
Paracetamol	52	40.3
Diclofenac	38	29.5
Piroxicam	26	20.2
Mefenamic acid	9	7.0
Aspirin	6	4.7
Indomethacin	3	2.3
Dynapar	3	2.3
Brustan	3	2.3
Tramadol	2	1.6
Herb	1	0.8
Meloxicam	1	0.8

### Prevalence of NSAID-related GI adverse effects

**Figure 2:**
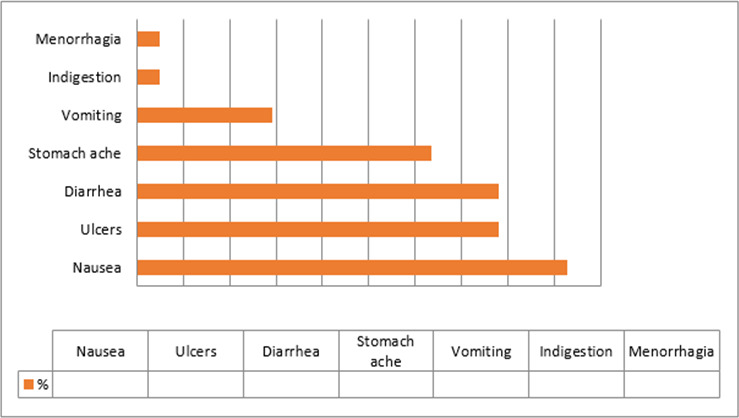
Frequency of NSAID-associated GI adverse effects among female students at Makerere University. GI, gastrointestinal; NSAID, non-steroidal anti-inflammatory drug.

Data from 129 female students who had used a medication while having dysmenorrhoea in the past 3 months were analysed. The prevalence of NSAID-associated GI adverse effects was 32% (n=41/129). Nausea (44%, n=18/41), ulcers (39%, n=16/41) and diarrhoea (39%, n=16/41) were the most frequently reported adverse, effects (figure 2). Ibuprofen (59%, n=24/41), diclofenac (46%, n=19/41), paracetamol (15%, n=6/41), indomethacin (7%, n=3/41) and aspirin (2%, n=1/41) were implicated for the adverse effects.

### Factors associated with NSAID-associated GI adverse effects

At multivariable logistic regression (table 3), factors independently associated with GI adverse effects were as follows: age at menarche (adjusted OR, AOR 1.57, 95% CI 1.05 to 2.34), the duration of menstruation (AOR 1.57, 95% CI 1.05 to 2.36) and use of ibuprofen (AOR 3.87, 95% CI 1.51 to 9.91). Females taking ibuprofen for dysmenorrhoea were about four times more likely to have NSAID-associated GIT adverse effects (AOR 3.87, 95% CI 1.51 to 9.91).

**Table 3:** Multivariable logistic regression showing factors associated with NSAID-associated GI adverse effects among female students at Makerere University

Variable	AOR	95% CI	P value
Current age	1.00	0.74 to 1.34	0.994
Age at menarche	1.57	1.05 to 2.34	0.026
Duration of menstruation	1.57	1.05 to 2.36	0.030
Regular menstrual periods			
Every time	1.00		
Most times	1.33	0.49 to 3.59	0.574
Sometimes	0.28	0.03 to 2.76	0.275
Rarely	5.87	0.37 to 93.35	0.210
Use of NSAID for pain relief			
Every time	1.00		
Most times	0.69	0.25 to 1.90	0.470
Sometimes	0.37	0.09 to 1.61	0.187
Ibuprofen	3.87	1.51 to 9.91	0.005

AOR, adjusted OR; GI, gastrointestinal; NSAID, non steroidal anti-inflammatory disease.

### Discontinuation for NSAID-associated GI adverse effects

Overall, 68% (88/314) of the female students reported having been advised to discontinue NSAIDs (table 4). Ulcers were the most common reason for discontinuation in 29 students (33%, 29/88). Of concern, some (8%, 7/88) of the students claimed that NSAIDs could cause infertility.

**Table 4:** Discontinuation of NSAID usage among female students at Makerere University

Variable	Frequency	%
Received advice to stop using a medication because of possible GI adverse effect		
Yes	88	68.0
No	41	32.0
Reason for advice to stop use of the NSAID?		
Ulcers	29	33.0
Infertility	7	8.0
Tolerance	3	3.4
Not good for women	2	2.3
Allergy	1	1.1
Can cause fibroids	1	1.1
Can cause cancer	1	1.1
Headache	1	1.1
Nausea	1	1.1
Menorrhagia	1	1.1
Vomiting	1	1.1
Nephrotic syndrome	1	1.1

GI, gastrointestinal; NSAID, non-steroidal anti-inflammatory drug.

## Discussion

This study aimed to determine the prevalence and factors associated with self-reported NSAIDs-related GI adverse effects among female students with dysmenorrhoea-associated pain at Makerere University. We found a high prevalence of GI adverse effects, reaching 32%. The factors significantly associated with adverse effects included age at menarche, duration of menstruation and ibuprofen use for pain relief.

Whereas there is a paucity of data on NSAID-related GI adverse effects among female university students, previous studies conducted in India, Pakistan and Japan among patients using NSAIDs reported a prevalence of GI adverse effects, such as peptic and gastric ulcers, estimated at 30.08%, 14.7% and 16.7%, respectively.^18–20^ In contrast to our findings, previous studies have shown that diclofenac was the most common NSAID associated with GI adverse effects.^18^ The difference could be attributed to variations in the most commonly used NSAID among participants in different studies and variations in study designs. A network meta-analysis by Feng and Wang revealed that tiaprofenic acid and mefenamic acid were indicated as the safest NSAIDs drugs, whereas indomethacin was the least safe with a higher likelihood to cause mild GI discomfort.^21^ In addition, a significant increase of about 2.5%–5.0% in the occurrence of GI adverse effects has been observed in individuals with a history of GI incidents.^22^

Comparable to findings from Ghana where the prevalence of medication use for dysmenorrhoea was 46%, our study observed that 47% (129/273) of participants used medications.^8^ However, the frequency of the use of medication to alleviate pain greatly varied among the participants and could be attributed to the intensity of pain and unregulated drug use as indicated by our finding that about 90% of participants acquired the painkillers over the counter at the pharmacy.^23^ Contrary to a study by Moore *et al* showed that ibuprofen was a much safer drug than paracetamol, our study showed that females taking ibuprofen for dysmenorrhoea were about four times more likely to have NSAID-associated GIT adverse effects (59%) than paracetamol (15%).^24^

Whereas our study deduced that 1-year increase in the age at menarche and 1-day increase in the duration of menstruation were 1.57 more likely associated with having NSAID-associated GIT adverse effects, other factors implicated in other studies include *Helicobacter pylori* infection, high-dose or multiple NSAID use, history of upper GI injury, receiving hemodialysis and anticoagulant, oral corticosteroid or selective serotonin reuptake inhibitor use.^25–28^

GI adverse effects are associated with increased physician visits, risk of GI surgery, high healthcare costs and poorer health-related quality of life.^29^ However, potential strategies such as the use of GI sparing NSAIDs, use of mucoprotective drugs and alternative medications or no pharmacological remedies for pain relief have been adopted in order to prevent NSAID-induced GI adverse effects.^30 31^

Our study contributes to the existing literature on the current status of NSAID-related GI effects among female university students in Uganda. However, there were some limitations. Due to the COVID-19 pandemic, the study was conducted online. Therefore, a limited number of students with internet access participated. This also limited the nature of study participant selection as we were unable to use probability sampling methods, the sampling technique used may not have given us the best representative study population. In addition, the exclusive use of a quantitative design in our study prevented us from exploring important perspectives that could have been derived from a qualitative study. While we performed statistical analyses to examine variable associations, we did not explicitly test for potential confounding and interaction effects, which may exist and have the potential to influence our findings.

## Conclusion

In this study, we found a considerably high prevalence of NSAID-related GI adverse effects associated with; age at menarche, longer duration of menstruation and ibuprofen use among female students at Makerere University who experienced dysmenorrhoea. Therefore, efforts directed towards mass education of females about the prevention, early identification and management of NSAID-related GI adverse effects are necessary. We recommend further exploration of alternative strategies for pain relief for females with dysmenorrhoea.

## Supplementary Material

Reviewer comments

Author's manuscript

## Data Availability

Data are available on reasonable request. If you wish to reuse any or all of this article, data are available on reasonable request.
