# Supplementary material for: Self-reported gastrointestinal adverse effects of non-steroidal anti-inflammatory drugs in female students with dysmenorrhoea at Makerere University: prevalence, discontinuation and associated factors. a cross sectional study
Source: BMJ Open. 2024 Jun 6;14(6):e079660. doi: 10.1136/bmjopen-2023-079660 (PMC11163621; doi:10.1136/bmjopen-2023-079660)
Supplement: Author's manuscript [file bmjopen-2023-079660.draft_revisions.pdf]

BMJ Open

BMJ Open is committed to open peer review. As part of this commitment we make the peer review history of every article we publish publicly available.

When an article is published we post the peer reviewers' comments and the authors' responses online. We also post the versions of the paper that were used during peer review. These are the versions that the peer review comments apply to.

The versions of the paper that follow are the versions that were submitted during the peer review process. They are not the versions of record or the final published versions. They should not be cited or distributed as the published version of this manuscript.

BMJ Open is an open access journal and the full, final, typeset and author-corrected version of record of the manuscript is available on our site with no access controls, subscription charges or pay-per-view fees (http://bmjopen.bmj.com).

If you have any questions on BMJ Open's open peer review process please email info.bmjopen@bmj.com

Self-reported gastrointestinal adverse effects of non-steroidal anti-inflammatory drugs in female university students with dysmenorrhea: Prevalence, discontinuation and associated factors

Journal:	*BMJ Open*
Manuscript ID	bmjopen-2023-079660
Article Type:	Original research
Date Submitted by the Author:	19-Sep-2023

Complete List of Authors:	Gobba, Solomon; Makerere University<br>Kibone, Winnie; Makerere University<br>Kiguba, Ronald; Makerere University

Keywords:	Dysmenorrhea, Adverse events < THERAPEUTICS, REPRODUCTIVE MEDICINE

SCHOLARONE™
Manuscripts

I, the Submitting Author has the right to grant and does grant on behalf of all authors of the Work (as defined in the below author licence), an exclusive licence and/or a non-exclusive licence for contributions from authors who are: i) UK Crown employees; ii) where BMJ has agreed a CC-BY licence shall apply, and/or iii) in accordance with the terms applicable for US Federal Government officers or employees acting as part of their official duties; on a worldwide, perpetual, irrevocable, royalty-free basis to BMJ Publishing Group Ltd ("BMJ") its licensees and where the relevant Journal is co-owned by BMJ to the co-owners of the Journal, to publish the Work in this journal and any other BMJ products and to exploit all rights, as set out in our licence.

The Submitting Author accepts and understands that any supply made under these terms is made by BMJ to the Submitting Author unless you are acting as an employee on behalf of your employer or a postgraduate student of an affiliated institution which is paying any applicable article publishing charge ("APC") for Open Access articles. Where the Submitting Author wishes to make the Work available on an Open Access basis (and intends to pay the relevant APC), the terms of reuse of such Open Access shall be governed by a Creative Commons licence – details of these licences and which Creative Commons licence will apply to this Work are set out in our licence referred to above.

Other than as permitted in any relevant BMJ Author's Self Archiving Policies, I confirm this Work has not been accepted for publication elsewhere, is not being considered for publication elsewhere and does not duplicate material already published. I confirm all authors consent to publication of this Work and authorise the granting of this licence.

For peer review only

Self-reported gastrointestinal adverse effects of non-steroidal anti-inflammatory drugs in female university students with dysmenorrhea: Prevalence, discontinuation and associated factors

Solomon Gobba[1], Winnie Kibone[2], Ronald Kiguba[3]

Affiliations
[1]School of Health Sciences, College of Health Sciences, Makerere University, Kampala, Uganda

[2]School of Medicine, College of Health Sciences, Makerere University, Kampala, Uganda

[3] School of Biomedical Sciences, College of Health Sciences, Makerere University, Kampala, Uganda

***Corresponding author**

Gobba Solomon
School of Health Sciences, College of Health Sciences, Makerere University, Kampala, Uganda
Email: solomongobba@gmail.com

Author details

Solomon Gobba: solomongobba@gmail.com
Winnie Kibone: kibonewinnie@gmail.com
Ronald Kiguba: kiguba@gmail.com

For peer review only

Abstract

Background: Non-Steroidal Anti-inflammatory Drugs (NSAIDS) are the most commonly used therapeutic remedies for dysmenorrhea in Uganda. However, NSAIDS are associated with a 3-to-5-fold increase in the risk of gastrointestinal (GI) adverse drug effects. We aimed to determine the prevalence and associated factors of self-reported NSAID-related GI adverse effects in female students who use NSAIDs in managing dysmenorrhea-associated pain at Makerere University.

Method: We conducted a cross-sectional study among female students at Makerere University, Kampala, Uganda from 14th October 2020 to 29th October 2020. Socio-demographic, menstrual history and treatment data were collected online using a pretested questionnaire, cleaned using Microsoft Excel 2016 and analyzed with STATA 15.1 software. Logistic regression was used to determine factors associated with self-reported adverse effects of NSAIDs among the female students.

Results: Overall, 314 valid responses were received from female students with a median age of 22 years (IQR: 18 – 29 years). The median age at menarche was 13 years (IQR: 9 – 18 years). 41% (n=129/314) of the respondents had used medication for dysmenorrhea, and 32% (n=41/129) of whom reported an NSAID-associated GI adverse effects with nausea being the most frequently reported (44%, n=18/41)

Factors independently associated with GI adverse effects were: age at menarche (p=0.026), duration of menstruation (P=0.030) and use of ibuprofen (p=0.005). Females taking ibuprofen for dysmenorrhoea were about 4 times as likely to have NSAID-associated GI adverse effects (AOR: 3.87, 95% CI: 1.51 – 9.91) than those who didn't receive ibuprofen.

Conclusion: We found a considerably high prevalence of NSAID-related GI adverse effects driven by factors such as age at menarche and ibuprofen use. Further exploration of alternative strategies for pain relief for females with dysmenorrhea is recommended.

For peer review only

Keywords: NSAIDs, gastrointestinal (GI), adverse effect, dysmenorrhea, Uganda.

Introduction

Dysmenorrhea is defined as painful menstrual cramps and is classified into primary dysmenorrhea without any evident pathology and secondary dysmenorrhea with a pathology (1, 2). Primary dysmenorrhea occurs in up to 50% of menstruating females and causes significant disruption in quality of life and absenteeism from school and work places affecting their productivity (3). The pain is attributed to uterine prostaglandins released by endometrial cells during uterine wall sloughing prior to the onset of menstruation (4). On account of the high prevalence of dysmenorrhea with a global prevalence ranging between 20% to 90%, dysmenorrhea is a serious public health burden (5). Studies have documented an equally higher prevalence of dysmenorrhea estimated at about 65.4%, 72.7% and 83.6% in Egypt, Ghana and Turkey respectively (6-8). In Uganda, the prevalence of dysmenorrhea has been estimated at 75.8% (9).

Non-steroidal anti-inflammatory drugs (NSAIDs) remain the most commonly used therapeutic remedies for dysmenorrhea in Uganda because of their affordability, convenience of use and efficacy (10). NSAIDs are commonly used as over-the-counter drugs and may often not be prescribed (11, 12). NSAIDs relieve symptoms in up to 70% of women when used correctly. Furthermore, NSAIDs are the recommended first-line treatment in management of dysmenorrhea unless there are contraindications such as history of hypersensitivity to aspirin or other NSAIDs, serious comorbidity, and GI ulcers or bleeding (4).

The utilization of NSAIDs has been linked to a significant 3- to 5-fold increase in the risk of GI adverse effects (13). Dyspepsia with pyrosis, abdominal pain, nausea, anorexia, gastric erosions, ulcers, perforation, GI hemorrhage which may result in anemia are some of the commonly observed GI adverse effects in NSAID therapy (14). Studies have explored GI complications of

For peer review only

NSAID-related treatment in cases of rheumatoid arthritis and osteoarthritis in the elderly (15) but little is known in relation to GI adverse drug effects related to NSAIDs therapy in the management of dysmenorrhea.

This study, aimed to determine the; 1) commonly used NSAIDs, 2) prevalence of self-reported NSAID-related GI adverse effects and, 3) factors associated with self-reported NSAID-related GI adverse effects during the management of dysmenorrhea among female students at Makerere University, Kampala, Uganda.

Methods

Study design

A descriptive cross-sectional study was conducted, between 14th October 2020 and 29th October 2020 to determine the prevalence and factors associated with GI adverse effects to NSAIDs usage in the management of dysmenorrhea among female students at Makerere University.

Study setting

The study was conducted at Makerere University main campus, situated North of Kampala, the capital city of Uganda, at the coordinates 0.3293° N, 32.5711° E. Makerere University is the largest public university in Uganda, attracting students from various regions of the country. The university accommodates over 40,000 students, both resident and non-resident. The main campus features twelve Halls of Residence, with six for male students and three for female students. Medical students are accommodated at the Mulago Hospital Complex. Approximately 5,000 resident undergraduates and 100 graduate students, accounting for 13% of the university's registered students, reside in the Halls of Residence. The remaining students either live in private

For peer review only

hostels or commute from their homes. The age range of the majority of students falls between 20 and 30 years, encompassing a diverse representation of socio-economic statuses.

Study population

Female students pursuing an undergraduate program at Makerere University and residing in different halls of residence and hostels.

The sample size of 310 participants with was calculated using the modified Kish and Leslie formula (16) for finite population size, with an estimated prevalence of dysmenorrhea among university students (75.8%) (17), and type 1 error of 5%, and a standard deviation at 95% confidence interval (1.96).

Selection criteria

All registered female students of Makerere University aged 18 years and older were eligible for the study after informed consent. Female students with primary or secondary amenorrhea, in menopause, with mental incapacitation or without access to internet were excluded from the study.

Data Collection

We used a self-administered questionnaire to collect data for this study. The questionnaire was pre-tested for comprehension and appropriateness among 15 students and adjusted accordingly to ensure quality data collection. The data were collected online via a Google survey document where participants received the survey information via a link shared with them in their WhatsApp groups and in their personal email inboxes. Eligible participants consented to the study and completed the survey. The submitted survey responses were automatically stored in an online data bank, accessible only to the Principal Investigator (PI) and the Supervisor. Additionally, the data were

For peer review only

backed up on the Principal Investigator's Google Drive to ensure data security. The questionnaire variables were constituted in three parts: sociodemographic information, menstrual history, and treatment variables. These sections aimed to gather comprehensive data relevant to the study objectives.

Study Variables

Independent variables: social demographics (age, and history of pregnancy), menstrual history (dysmenorrhea, menarche, regularity and duration of menstrual periods) and treatment (painkillers used and their source, side effects).

Dependent variables: NSAID-related GI adverse effects.

Data quality control

To ensure that high quality data were captured from the volunteers, the data collection tool was pretested and adjusted to ensure convenience and ease of manoeuvre. Checks were incorporated within the document to ensure all questions were responded to and correctly answered to reduce the margins of error.

Data management

Data collection tools were checked for completeness and instantly submitted to a central data bank that tallied all responses received from the participants. The central databank was synchronized and backed up on the PI's google drive for secondary storage. Upon completion of the survey, the data were translated into Excel for data cleaning and initial analysis. The data were then exported to STATA Software (StataCorp LLC, USA) for further analysis.

For peer review only

Data analysis

All analyses were performed using Microsoft Excel 2016 and STATA Software. Numerical data were summarized as means and standard deviations if normally distributed; otherwise, median and interquartile ranges were used for non-normally distributed data. Categorical data were summarized as frequencies and proportions with their 95% confidence intervals. Associations between independent variables and dependent variables were assessed using the chi-square test and multivariable analysis using STATA 15.1 software. A P-Value of less than 0.05 was considered statistically significant.

Ethical and Regulatory Approval

We obtained ethical approval from of the School of Biomedical Sciences Research and Ethics Committee, Makerere University prior to study implementation, REC Approval Number SBS-REC -888. Participation in the study was voluntary and participants provided informed consent prior to enrolment into the study. All information was kept confidential and used for research purposes only.

Results

Characteristics of Participants

Overall, a total of 314 participants responded to the study. The median age was 22 years (IQR: 21-23). The majority came from nuclear families (62%, 194/314). Only 8% (26/314) of the participants had a history of pregnancy. The median age at menarche was 13 years (IQR: 12-14) with range of 9 to 18 years and more than half (55%, 173/314) experienced regular periods every

For peer review only

time. Nearly all female students were aware of dysmenorrhoea (99%, 310/314). **Table 1** summarizes the baseline characteristics of participants.

Table 1. Demographic and Menstrual Characteristics of Participants

Variable	Frequency (n)	Percentage (%)
Age of respondents, years (Median, IQR)	22	21-23
18-21	70	22.3
22-25	168	53.5
26-29	40	12.7
30 and above	36	11.5
Age at menarche, years (median, IQR)	13	12-14
9-11	24	7.6
12-14	206	65.6
15 and above	84	26.8
Type of family		
Nuclear	194	61.8
Extended	81	25.8
Single parent	37	11.8
Divorced parents	2	0.6
History of pregnancy		
Yes	26	8.3
No	288	91.7
Awareness on dysmenorrhoea		
Yes	310	98.7
No	4	1.3
Regular menstrual periods		
Every time	173	55.1
Most times	91	29.0
Sometimes	34	10.8
Rarely	16	5.1
Duration of menstrual periods (days)		
1-2	10	3.2
3-4	95	30.3
5-6	76	24.2
7-8	19	6.1
9 and above	1	0.3

For peer review only

Patterns of dysmenorrhea and medication use

The majority of participants (42%, 132/314) reported experiencing menstrual cramps every time and about a quarter (23%, 71/314) reported using medicines to reduce pain during menstrual pain every time. Most (87%, 273/314) of the participants reported experiencing menstrual cramps in the past three months of whom nearly half (47%, 129/273) reported using a medication for menstrual cramps with Ibuprofen and paracetamol being the most frequently used medications (40%, 52/129 and 40%, 52/129 respectively). The majority (90%, 116/129) bought the medication from pharmacies. **Supplementary Figure 1** shows the sources of the medication. **Table 2** summarizes the patterns of dysmenorrhea and medication use among female students at Makerere University.

Table 2. Patterns of dysmenorrhoea and medication use among female students at Makerere University

Variable	Frequency	%
Experienced menstrual pains/ cramps/ dysmenorrhea		
Every time	132	42.0
Most times	71	22.6
Sometimes	76	24.2
Rarely	35	11.2
Frequency of use of medicines (pain killers) to reduce pain during your menstrual period		
Every time	71	22.6
Most times	52	16.6
Sometimes	60	19.1
Rarely	131	41.7
Experienced menstrual cramps in the past 3 months		
Yes	273	86.9
No	41	13.1
How long ago the most recent period was (days)		
1-5	180	57.32
6-10	98	31.21
11 and above	36	11.47

For peer review only

Use of medication		
Yes	129	47.3
No	144	52.8
Medicines used for menstrual pains/cramps/ dysmenorrhea		
Ibuprofen	52	40.3
Paracetamol	52	40.3
Diclofenac	38	29.5
Piroxicam	26	20.2
Mefenamic Acid	9	7.0
Aspirin	6	4.7
Indomethacin	3	2.3
Dynapar	3	2.3
Brustan	3	2.3
Tramadol	2	1.6
Herb	1	0.8
Meloxicam	1	0.8

Prevalence of NSAID-Related GI adverse effects

Data of 129 female students who had used a medication while having dysmenorrhoea in the past three months were analysed. The prevalence of NSAID-associated GI adverse effects was 32% (n=41/129). Nausea (44%, n=18/41), ulcers (39%, n=16/41) and diarrhoea (39%, n=16/41) were the most frequently reported adverse effects. **Supplementary Figure 2** shows the frequency of NSAID-associated GI adverse effects. Ibuprofen (59%, n=24/41), diclofenac (46%, n=19/41), paracetamol (15%, n=6/41), indomethacin (7%, n=3/41) and aspirin (2%, n=1/41) were implicated for the adverse effects.

Factors associated with NSAID-associated gastrointestinal adverse effects

At multivariable logistic regression, **Table 3**. Factors independently associated with GI adverse effects were: age at menarche (AOR: 1.57, 95% CI: 1.05 - 2.34), duration of menstruation (AOR:

For peer review only

1.57, 95% CI: 1.05 - 2.36). and use of ibuprofen (AOR: 3.87, 95% CI: 1.51 - 9.91). Females taking ibuprofen for dysmenorrhoea were about 4 times more likely to have NSAID-associated GIT adverse effects (AOR: 3.87, 95% CI: 1.51 – 9.91).

Table 3. Multivariable logistic regression showing factors associated with NSAID-associated GI adverse effects among female students at Makerere University

**Variable**	**AOR**	**95% CI**	**P**
Current age	1.00	0.74 - 1.34	0.994
Age at menarche	1.57	1.05 - 2.34	0.026
Duration of menstruation	1.57	1.05 - 2.36	0.030
Regular menstrual periods			
Every time	1.00		
Most times	1.33	0.49 - 3.59	0.574
Sometimes	0.28	0.03 - 2.76	0.275
Rarely	5.87	0.37 - 93.35	0.210
Use of NSAID for pain relief			
Every time	1.00		
Most times	0.69	0.25 - 1.90	0.470
Sometimes	0.37	0.09 - 1.61	0.187
Ibuprofen	3.87	1.51 - 9.91	0.005

Note: AOR- adjusted odds ratio, CI- confidence interval, NSAID- non steroidal anti-inflammatory disease

For peer review only

Discontinuation for NSAID-associated GI adverse effects

Overall, 68% (88/314) of the female students reported having been advised to discontinue NSAIDs (**Table 4**). Ulcers were the most common reason for discontinuation in 29 students (33%, 29/88). Of concern, some (8%, 7/88) of the students claimed that NSAIDs could cause infertility.

Table 4. Discontinuation of NSAID usage among female students at Makerere University

Variable	Frequency	%
Received advice to stop using a medication because of possible GI adverse effect		
Yes	88	68.0
No	41	32.0
Reason for advice to stop use of the NSAID?		
Ulcers	29	33.0
Infertility	7	8.0
Tolerance	3	3.4
Not good for women	2	2.3
Allergy	1	1.1
Can cause fibroids	1	1.1
Can cause cancer	1	1.1
Headache	1	1.1
Nausea	1	1.1
Menorrhagia	1	1.1
Vomiting	1	1.1
Nephrotic syndrome	1	1.1

For peer review only

Discussion

This study, aimed to determine the prevalence and factors associated with self-reported non-steroidal anti-inflammatory drugs (NSAIDs)-related gastrointestinal (GI) adverse effects among female students with dysmenorrhea-associated pain at Makerere University. We found a high prevalence of GI adverse effects, reaching 32%. The factors significantly associated with adverse effects included age at menarche, duration of menstruation, and ibuprofen use for pain relief.

Whereas there is a paucity of data regarding GI adverse effects among female university students, previous studies conducted in India, Pakistan and Japan among patients using NSAIDs reported a prevalence of GI adverse effects, such as peptic and gastric ulcers, estimated at 30.08%, 14.7% and 16.7% respectively (18-20). In contrast to our findings, previous studies have showed that diclofenac was the commonest NSAID associated with GI adverse effects (18). The difference could be attributed to variations in the most commonly used NSAID among participants in different studies and variations in study designs. In addition, a significant increase of about 2.5-5.0% in the occurrence of GI adverse effects has been observed in individuals with a history of GI incidents (21).

Comparable to findings from Ghana where the prevalence of medication use for dysmenorrhea was 46%, our study observed that 47% (129/273) of participants used medications (22). However, the frequency of the use of medication to alleviate pain greatly varied amongst the participants and could be attributed to the intensity of pain and unregulated drug use as indicated by our finding that about 90% of participants acquired the painkillers over the counter at the pharmacy (23). Contrary to a study by Moore and colleagues that showed that ibuprofen was a much safer drug than paracetamol, our study showed that females taking ibuprofen for dysmenorrhoea were about 4 times more likely to have NSAID-associated GIT adverse effects (59%) than paracetamol (15%) (24).

Whereas our study deduced that 1-year increase in the age at menarche and one-day increase in the duration of menstruation were 1.57 more likely associated with having NSAID-associated GIT adverse effects, other factors implicated in other studies include; *Helicobacter pylori* infection, high dose or multiple NSAID use, history of upper GI injury, receiving hemodialysis and anticoagulant, oral corticosteroid or selective serotonin reuptake inhibitor use (25-28).

For peer review only

GI adverse effects are associated with increased physician visits, risk of GI surgery, high health care costs and poorer health related quality of life (HRQoL) (29). However, potential strategies such as the use of GI sparing NSAIDs, use of mucoprotective drugs and alternative medications or no pharmacological remedies for pain relief have been adopted in order to prevent NSAID induced GI adverse effects (30, 31).

Our study contributes to the existing literature on the current status of NSAID-related GI effects among female university students in Uganda. However, there were some limitations. Due to the COVID-19 pandemic, the study was conducted online. Therefore, a limited number of students with internet access participated. This also limited the nature of study participant selection as we were unable to use probability sampling methods. In addition, the exclusive use of a quantitative design in our study prevented us from exploring important perspectives that could have been derived from a qualitative study. While we performed statistical analyses to examine variable associations, we did not explicitly test for potential confounding and interaction effects, which may exist and have the potential to influence our findings.

Conclusion

In this study, we found a considerably high prevalence of NSAID-related GI adverse effects associated with; age at menarche, longer duration of menstruation, and ibuprofen use among female students at Makerere University experienced dysmenorrhea. Therefore, efforts directed towards mass education of females about the prevention, early identification and management of NSAID-related GI adverse effects are necessary. We recommend further exploration of alternative strategies for pain relief for females with dysmenorrhea.

For peer review only

Acknowledgments

The authors acknowledge the contribution of the following team of individuals in the data collection process. The team is as follows; Dr. Haruna Kiryowa, Mr. David Bakka, Mr. Gerald Akena, Mr. Samuel Ssenkungu, Ms. Catherine Aiya Lalam and Ms. Gillian Sheila Amuge.

Contributorship statement

All authors contributed significantly to study conceptualisation, data collection, analysis, interpretation, and manuscript writing. All authors read and approved the final manuscript.

Competing interests

All authors declare that they have no conflict of interest.

Patient and public involvement

Patients and/or the public were not involved in the design, or conduct or reporting or dissemination plans of this research.

Funding

The Medical Research Council (MR/V03510X/1) gave financial support to facilitate the writing of this manuscript.

Data sharing statement

No additional data are available.

For peer review only

References

1. Kumar KH, Elavarasi PJJoAC, Insights R. Definition of pain and classification of pain disorders. 2016;3(3):87-90.
2. Smith RP. The clinical classification and causes of dysmenorrhea. Dysmenorrhea and Menorrhagia: Springer; 2018. p. 55-64.
3. Dawood MY. Primary dysmenorrhea: advances in pathogenesis and management. Obstetrics & Gynecology. 2006;108(2):428-41.
4. Proctor MML, Farquhar CM. Dysmenorrhoea. BMJ clinical evidence. 2007;2007.
5. Vincenzo De Sanctis M, Soliman A, Bernasconi S, Bianchin L, Bona G, Bozzola M, et al. Primary dysmenorrhea in adolescents: prevalence, impact and recent knowledge. 2015;13(2):465-73.
6. Unsal A, Ayranci U, Tozun M, Arslan G, Calik EJUjoms. Prevalence of dysmenorrhea and its effect on quality of life among a group of female university students. 2010;115(2):138-45.
7. Ameade EPK, Amalba A, Mohammed BSJBWsH. Prevalence of dysmenorrhea among University students in Northern Ghana; its impact and management strategies. 2018;18(1):1-9.
8. Nooh AMJMEFSJ. Menstrual disorders among Zagazig university students, Zagazig, Egypt. 2015;20(3):198-203.
9. Nakame RM, Kiwanuka F, Robert AJNo. Dysmenorrhoea among students aged 18–45 years attending University in Uganda: A cross-sectional multicenter study of three Universities in Uganda. 2019;6(2):268-75.
10. Alshakka MA, Badullah WF, Alolayan SO, Mahmoud MAJBR. Prescribing patterns of non-steroidal anti-inflammatory drugs (NSAIDs) at outpatient departments of four hospitals. 2018;29(19):3643-7.
11. Duong M, Salvo F, Pariente A, Abouelfath A, Lassalle R, Droz C, et al. Usage patterns of 'over-the-counter'vs. prescription-strength nonsteroidal anti-inflammatory drugs in F rance. 2014;77(5):887-95.
12. Wilcox CM, Cryer B, Triadafilopoulos GJTJor. Patterns of use and public perception of over-the-counter pain relievers: focus on nonsteroidal antiinflammatory drugs. 2005;32(11):2218-24.
13. Castellsague J, Riera-Guardia N, Calingaert B, Varas-Lorenzo C, Fourrier-Reglat A, Nicotra F, et al. Individual NSAIDs and upper gastrointestinal complications. Drug safety. 2012;35(12):1127-46.
14. Vostinaru O. Adverse Effects and Drug Interactions of the Non-Steroidal Anti-Inflammatory Drugs. Nonsteroidal Anti-Inflammatory Drugs. 2017:17.
15. MacDonald T. Epidemiology and pharmacoeconomic implications of non-steroidal anti-inflammatory drug-associated gastrointestinal toxicity. Rheumatology. 2000;39(suppl_2):13-20.
16. Kish L. Survey sampling. John Wiley and Sons, Inc, New York. 1965.
17. Nakame RM. Factors Influencing the Prevalence of Dysmenorrhea Among Students Aged 18-45 Years Attending Institutions of Higher Learning in Kampala Capital City: International Health Sciences University.; 2015.
18. Hamid S, Yakoob J, Jafri W, Islam S, Abid S, Islam MJJoPMA. Frequency of NSAID induced peptic ulcer disease. 2006;56(5):218.
19. Chatterjee S, Dureja GP, Kadhe G, Mane A, Phansalkar AA, Sawant S, et al. Cross-sectional study for prevalence of non-steroidal anti-inflammatory drug-induced gastrointestinal, cardiac and renal complications in India: interim report. 2015;8(3-4):216.

For peer review only

20. Tsujimoto S, Mokuda S, Matoba K, Yamada A, Jouyama K, Murata Y, et al. The prevalence of endoscopic gastric mucosal damage in patients with rheumatoid arthritis. 2018;13(7):e0200023.
21. Dubois R, Melmed G, Henning J, Laine LJAp, therapeutics. Guidelines for the appropriate use of non-steroidal anti-inflammatory drugs, cyclo-oxygenase-2-specific inhibitors and proton pump inhibitors in patients requiring chronic anti-inflammatory therapy. 2004;19(2):197-208.
22. Ameade EPK, Amalba A, Mohammed BS. Prevalence of dysmenorrhea among University students in Northern Ghana; its impact and management strategies. BMC women's health. 2018;18(1):39.
23. Samba Conney C, Akwo Kretchy I, Asiedu-Danso M, Allotey-Babington GLJO, International G. Complementary and alternative medicine use for primary dysmenorrhea among senior high school students in the western region of Ghana. 2019;2019.
24. Moore NJIjocpS. Forty years of ibuprofen use. 2003(135):28-31.
25. Goldstein JL, Cryer BJD, healthcare, safety p. Gastrointestinal injury associated with NSAID use: a case study and review of risk factors and preventative strategies. 2015;7:31.
26. Jankovic SM, Aleksic J, Rakovic S, Aleksic A, Stevanovic I, Stefanovic-Stoimenov N, et al. Nonsteroidal antiinflammatory drugs and risk of gastrointestinal bleeding among patients on hemodialysis. 2009;22(4):502.
27. García Rodríguez LA, Hernández-Díaz SJAR, Therapy. The risk of upper gastrointestinal complications associated with nonsteroidal anti-inflammatory drugs, glucocorticoids, acetaminophen, and combinations of these agents. 2001;3(2):1-4.
28. Shorr RI, Ray WA, Daugherty JR, Griffin MRJAoim. Concurrent use of nonsteroidal anti-inflammatory drugs and oral anticoagulants places elderly persons at high risk for hemorrhagic peptic ulcer disease. 1993;153(14):1665-70.
29. Chang LJAp, therapeutics. Epidemiology and quality of life in functional gastrointestinal disorders. 2004;20:31-9.
30. Guo C-G, Leung WKJG, liver. Potential strategies in the prevention of nonsteroidal anti-inflammatory drugs-associated adverse effects in the lower gastrointestinal tract. 2020;14(2):179.
31. García-Rayado G, Navarro M, Lanas AJErocp. NSAID induced gastrointestinal damage and designing GI-sparing NSAIDs. 2018;11(10):1031-43.

For peer review only

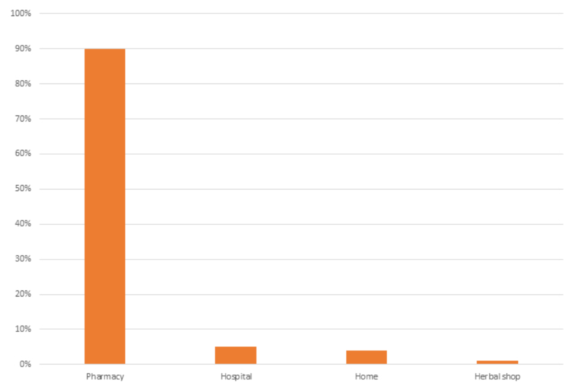

338x190mm (96 x 96 DPI)

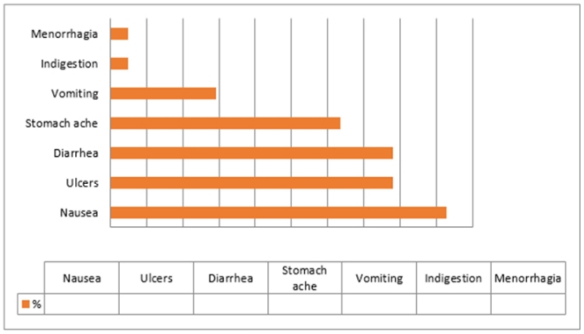

338x190mm (96 x 96 DPI)

STROBE Statement—Checklist of items that should be included in reports of ***cross-sectional studies***

	Item No	Recommendation
**Title and abstract**	1	(*a*) Indicate the study's design with a commonly used term in the title or the abstract-Page 1
		(*b*) Provide in the abstract an informative and balanced summary of what was done and what was found-Page 2
**Introduction**		
Background/rationale	2	Explain the scientific background and rationale for the investigation being reported-Page 3, 4
Objectives	3	State specific objectives, including any prespecified hypotheses-Page 4
**Methods**		
Study design	4	Present key elements of study design early in the paper-Page 4
Setting	5	Describe the setting, locations, and relevant dates, including periods of recruitment, exposure, follow-up, and data collection-Page 4,5
Participants	6	(*a*) Give the eligibility criteria, and the sources and methods of selection of participants-Page 5
Variables	7	Clearly define all outcomes, exposures, predictors, potential confounders, and effect modifiers. Give diagnostic criteria, if applicable-Page 6
Data sources/ measurement	8*	For each variable of interest, give sources of data and details of methods of assessment (measurement). Describe comparability of assessment methods if there is more than one group-Page 5
Bias	9	Describe any efforts to address potential sources of bias-Page 5
Study size	10	Explain how the study size was arrived at- page 5
Quantitative variables	11	Explain how quantitative variables were handled in the analyses. If applicable, describe which groupings were chosen and why-Page 6
Statistical methods	12	(*a*) Describe all statistical methods, including those used to control for confounding-Page 7
		(*b*) Describe any methods used to examine subgroups and interactions-Page 7
		(*c*) Explain how missing data were addressed-Page 7
		(*d*) If applicable, describe analytical methods taking account of sampling strategy-Page 7
		(*e*) Describe any sensitivity analyses-Page 7
**Results**		
Participants	13*	(a) Report numbers of individuals at each stage of study—eg numbers potentially eligible, examined for eligibility, confirmed eligible, included in the study, completing follow-up, and analysed-Page 7
Descriptive data	14*	(a) Give characteristics of study participants (eg demographic, clinical, social) and information on exposures and potential confounders-Page 7,8
		(b) Indicate number of participants with missing data for each variable of interest-Page 7
Outcome data	15*	Report numbers of outcome events or summary measures-Page 9
Main results	16	(*a*) Give unadjusted estimates and, if applicable, confounder-adjusted estimates and their precision (eg, 95% confidence interval). Make clear which confounders were adjusted for and why they were included-Page 12, 13

For peer review only - http://bmjopen.bmj.com/site/about/guidelines.xhtml

Other analyses	17	Report other analyses done—eg analyses of subgroups and interactions, and sensitivity analyses-Page 13
**Discussion**		
Key results	18	Summarise key results with reference to study objectives-page 14
Limitations	19	Discuss limitations of the study, taking into account sources of potential bias or imprecision. Discuss both direction and magnitude of any potential bias-Page 15
Interpretation	20	Give a cautious overall interpretation of results considering objectives, limitations, multiplicity of analyses, results from similar studies, and other relevant evidence-Page 14, 15
Generalisability	21	Discuss the generalisability (external validity) of the study results-Page 14, 15
**Other information**		
Funding	22	Give the source of funding and the role of the funders for the present study and, if applicable, for the original study on which the present article is based-Page 16

For peer review only

BMJ Open

Self-reported gastrointestinal adverse effects of non-steroidal anti-inflammatory drugs in female university students with dysmenorrhea: Prevalence, discontinuation and associated factors

Journal:	*BMJ Open*
Manuscript ID	bmjopen-2023-079660.R1
Article Type:	Original research
Date Submitted by the Author:	07-Mar-2024
Complete List of Authors:	Gobba, Solomon; Makerere University<br>Kibone, Winnie; Makerere University<br>Kiguba, Ronald; Makerere University College of Health Sciences, Pharmacology and Therapeutics
**Primary Subject Heading**:	Pharmacology and therapeutics
Secondary Subject Heading:	Reproductive medicine
Keywords:	Dysmenorrhea, Adverse events < THERAPEUTICS, REPRODUCTIVE MEDICINE

SCHOLARONE™
Manuscripts

I, the Submitting Author has the right to grant and does grant on behalf of all authors of the Work (as defined in the below author licence), an exclusive licence and/or a non-exclusive licence for contributions from authors who are: i) UK Crown employees; ii) where BMJ has agreed a CC-BY licence shall apply, and/or iii) in accordance with the terms applicable for US Federal Government officers or employees acting as part of their official duties; on a worldwide, perpetual, irrevocable, royalty-free basis to BMJ Publishing Group Ltd ("BMJ") its licensees and where the relevant Journal is co-owned by BMJ to the co-owners of the Journal, to publish the Work in this journal and any other BMJ products and to exploit all rights, as set out in our licence.

The Submitting Author accepts and understands that any supply made under these terms is made by BMJ to the Submitting Author unless you are acting as an employee on behalf of your employer or a postgraduate student of an affiliated institution which is paying any applicable article publishing charge ("APC") for Open Access articles. Where the Submitting Author wishes to make the Work available on an Open Access basis (and intends to pay the relevant APC), the terms of reuse of such Open Access shall be governed by a Creative Commons licence – details of these licences and which Creative Commons licence will apply to this Work are set out in our licence referred to above.

Other than as permitted in any relevant BMJ Author's Self Archiving Policies, I confirm this Work has not been accepted for publication elsewhere, is not being considered for publication elsewhere and does not duplicate material already published. I confirm all authors consent to publication of this Work and authorise the granting of this licence.

For peer review only

Self-reported gastrointestinal adverse effects of non-steroidal anti-inflammatory drugs in female university students with dysmenorrhea: Prevalence, discontinuation and associated factors

Solomon Gobba[1], Winnie Kibone[2], Ronald Kiguba[3]

Affiliations

[1]School of Health Sciences, College of Health Sciences, Makerere University, Kampala, Uganda

[2]School of Medicine, College of Health Sciences, Makerere University, Kampala, Uganda

[3] School of Biomedical Sciences, College of Health Sciences, Makerere University, Kampala, Uganda

***Corresponding author**

Gobba Solomon
School of Health Sciences, College of Health Sciences, Makerere University, Kampala, Uganda
Email: solomongobba@gmail.com

Author details

Solomon Gobba: solomongobba@gmail.com
Winnie Kibone: kibonewinnie@gmail.com
Ronald Kiguba: kiguba@gmail.com

For peer review only

Abstract

Background: Primary dysmenorrhea occurs in up to 50% of menstruating females. Non-Steroidal Anti-inflammatory Drugs (NSAIDS) are the most commonly used therapeutic remedies for dysmenorrhea in Uganda. However, NSAIDS are associated with a 3-to-5-fold increase in the risk of gastrointestinal (GI) adverse drug effects. We aimed to determine the prevalence and associated factors of self-reported NSAID-related GI adverse effects in female students who use NSAIDs in managing dysmenorrhea-associated pain at Makerere University.

Method: We conducted a cross-sectional study among female students at Makerere University, Kampala, Uganda from 14th October 2020 to 29th October 2020. Socio-demographic, menstrual history and treatment data were collected online using a pretested questionnaire, cleaned using Microsoft Excel 2016 and analyzed with STATA 15.1 software. Logistic regression was used to determine factors associated with self-reported adverse effects of NSAIDs among the female students. $p < 0.05$ was considered statistically significant.

Results: Overall, 314 valid responses were received from female students with a median age of 22 years (IQR: 18 – 29 years). The median age at menarche was 13 years (IQR: 9 – 18 years). 41% (n=129/314) of the respondents had used medication for dysmenorrhea, and 32% (n=41/129) of whom reported an NSAID-associated GI adverse effects with nausea being the most frequently reported (44%, n=18/41)

Factors independently associated with GI adverse effects were: age at menarche (p=0.026), duration of menstruation (P=0.030) and use of ibuprofen (p=0.005). Females taking ibuprofen for dysmenorrhoea were about 4 times as likely to have NSAID-associated GI adverse effects (AOR: 3.87, 95% CI: 1.51 – 9.91) than those who didn’t receive ibuprofen.

Conclusion: We found a considerably high prevalence of NSAID-related GI adverse effects driven by factors such as age at menarche and ibuprofen use.

For peer review only

Keywords: NSAIDs, gastrointestinal (GI)**,** adverse effect, dysmenorrhea, Uganda.

Strengths and Limitations of the study

The study was conducted online due to the COVID-19 pandemic hence a limited number of students with internet access participated.

Based on the nature of the data collection, it was also impossible to determine how many students were reached hence impossible to determine a response rate.

This also limited the nature of study participant selection as we were unable to use probability sampling methods.

The study was primarily quantitative and this prevented us from exploring important perspectives that could have been derived from a qualitative study.

Introduction

Dysmenorrhea is defined as painful menstrual cramps and is classified into primary dysmenorrhea without any evident pathology and secondary dysmenorrhea with a pathology (1, 2). Pinpointing the exact prevalence remains elusive, as studies conducted in diverse populations show varying rates, spanning from 20% to 94% (3) Primary dysmenorrhea occurs in up to 50% of menstruating females and causes significant disruption in quality of life and absenteeism from school and work places affecting their productivity (4). The pain is attributed to uterine prostaglandins released by endometrial cells during uterine wall sloughing prior to the onset of menstruation (5). On account of the high prevalence of dysmenorrhea with a global prevalence ranging between 20% to 90%, dysmenorrhea is a serious public health burden (6). Studies have documented an equally higher prevalence of dysmenorrhea estimated at about 65.4%, 72.7% and 83.6% in Egypt, Ghana and Turkey respectively (7-9). In Uganda, the prevalence of dysmenorrhea has been estimated at 75.8% (10).

For peer review only

Non-steroidal anti-inflammatory drugs (NSAIDs) remain the most commonly used therapeutic remedies for dysmenorrhea in Uganda because of their affordability, convenience of use and efficacy (11). NSAIDs are commonly used as over-the-counter drugs and may often not be prescribed (12, 13). NSAIDs relieve symptoms in up to 70% of women when used correctly. Furthermore, NSAIDs are the recommended first-line treatment in management of dysmenorrhea unless there are contraindications such as history of hypersensitivity to aspirin or other NSAIDs, serious comorbidity, and GI ulcers or bleeding (5).

The utilization of NSAIDs has been linked to a significant 3- to 5-fold increase in the risk of GI adverse effects (14). Dyspepsia with pyrosis, abdominal pain, nausea, anorexia, gastric erosions, ulcers, perforation, GI hemorrhage which may result in anemia are some of the commonly observed GI adverse effects in NSAID therapy (15). Studies have explored GI complications of NSAID-related treatment in cases of rheumatoid arthritis and osteoarthritis in the elderly (16) but little is known in relation to GI adverse drug effects related to NSAIDs therapy in the management of dysmenorrhea. Furthermore, university students practice more self-medication using NSAIDs for dysmenorrhea. In fact, recent publication showed a high prevalence of self-medication estimated at 37% commonly mefenamic acid (58%).

This study, aimed to determine the commonly used NSAIDs, the prevalence of self-reported NSAID-related GI adverse effects and the factors associated with self-reported NSAID-related GI adverse effects during the management of dysmenorrhea among female students at Makerere University, Kampala, Uganda.

Methods

Study design

For peer review only

A descriptive cross-sectional study was conducted, between 14th October 2020 and 29th October 2020 to determine the prevalence and factors associated with GI adverse effects to NSAIDs usage in the management of dysmenorrhea among female students at Makerere University.

Study setting

The study was conducted at Makerere University main campus, situated North of Kampala, the capital city of Uganda, at the coordinates 0.3293° N, 32.5711° E. Makerere University is the largest public university in Uganda, attracting students from various regions of the country. The university accommodates over 40,000 students, both resident and non-resident. The main campus features twelve Halls of Residence, with six for male students and three for female students. Medical students are accommodated at the Mulago Hospital Complex. Approximately 5,000 resident undergraduates and 100 graduate students, accounting for 13% of the university's registered students, reside in the Halls of Residence. The remaining students either live in private hostels or commute from their homes. The age range of the majority of students falls between 20 and 30 years, encompassing a diverse representation of socio-economic statuses.

Study population

Female students pursuing an undergraduate program at Makerere University and residing in different halls of residence and hostels. The sample size of 314 participants was calculated using the modified Kish and Leslie formula(17) for finite population size, with an estimated prevalence of dysmenorrhea among university students (75.8%) , and a 10% adjustment was made to cater for nonresponse.

Selection criteria

For peer review only

129 All registered female students of Makerere University aged 18 years and older were eligible for
130 the study after informed consent. Purposive sampling technique was used to select students into
131 the study. Female students with primary or secondary amenorrhea, in menopause, with mental
132 incapacitation or without access to internet were excluded from the study.

133 **Data Collection**

134 We used a self-administered questionnaire to collect data for this study. The questionnaire was
135 pre-tested for comprehension and appropriateness among 15 students and adjusted accordingly to
136 ensure quality data collection. The data were collected online via a Google survey document where
137 participants received the survey information via a link shared with them in their WhatsApp groups
138 and in their personal email inboxes. Eligible participants consented to the study and completed the
139 survey. The submitted survey responses were automatically stored in an online data bank,
140 accessible only to the Principal Investigator (PI) and the Supervisor. Additionally, the data were
141 backed up on the Principal Investigator's Google Drive to ensure data security. The questionnaire
142 variables were constituted in three parts: sociodemographic information, menstrual history, and
143 treatment variables. These sections aimed to gather comprehensive data relevant to the study
144 objectives.

145 **Study Variables**

146 Independent variables: social demographics (age, and history of pregnancy), menstrual history
147 (dysmenorrhea, menarche, regularity and duration of menstrual periods) and treatment (painkillers
148 used and their source, side effects).

For peer review only

Dependent variables: NSAID-related GI adverse effects.

Data quality control

To ensure that high quality data were captured from the volunteers, the data collection tool was pretested and adjusted to ensure convenience and ease of manoeuvre. Checks were incorporated within the document to ensure all questions were responded to and correctly answered to reduce the margins of error.

Data management

Data collection tools were checked for completeness and instantly submitted to a central data bank that tallied all responses received from the participants. The central databank was synchronized and backed up on the PI's google drive for secondary storage. Upon completion of the survey, the data were translated into Excel for data cleaning and initial analysis. The data were then exported to STATA Software (StataCorp LLC, USA) for further analysis.

Data analysis

All analyses were performed using Microsoft Excel 2016 and STATA Software. Numerical data were summarized as means and standard deviations if normally distributed; otherwise, median and interquartile ranges were used for non-normally distributed data. Categorical data were summarized as frequencies and proportions with their 95% confidence intervals. Associations between independent variables and dependent variables were assessed using the chi-square test and multivariable analysis using STATA 15.1 software. A P-Value of less than 0.05 was considered statistically significant.

For peer review only

169 **Ethical and Regulatory Approval**

170 We obtained ethical approval from of the School of Biomedical Sciences Research and Ethics
171 Committee, Makerere University prior to study implementation, REC Approval Number SBS-
172 REC -888. Participation in the study was voluntary and participants provided informed consent
173 prior to enrolment into the study. All information was kept confidential and used for research
174 purposes only.

175 **Patient and Public Involvement**

176 Patients and the public were at the centre of this research, our research questions and outcome
177 measures were shaped by findings from previous studies which often reflect patient-reported
178 outcomes, adverse events, and medication-related errors. We aimed to develop research objectives
179 that were meaningful, relevant, and actionable in addressing medication safety issues from a
180 patient-centered standpoint.

181 We conducted a questionnaire pretest session with a select group of participants to refine study
182 questionnaire which ensured that the study design was sensitive to patient preferences and
183 experiences.

184 The results of the study will be disseminated to study participants and the general public through
185 various channels to ensure accessibility and comprehension. This will include the distribution of
186 lay summaries written in plain language, presentations at patient-focused conferences, and
187 publication of study findings in open-access journals.

188

189

190

For peer review only

Results

Characteristics of Participants

Overall, a total of 314 participants responded to the study. The median age was 22 years (IQR: 21-23). The majority came from nuclear families (62%, 194/314). Only 8% (26/314) of the participants had a history of pregnancy. The median age at menarche was 13 years (IQR: 12-14) with range of 9 to 18 years and more than half (55%, 173/314) experienced regular periods every time. Nearly all female students were aware of dysmenorrhoea (99%, 310/314). **Table 1** summarizes the baseline characteristics of participants.

Table 1. Demographic and Menstrual Characteristics of Participants

**Variable**	**Frequency (n)**	**Percentage (%)**
Age of respondents, years (Median, IQR)	22	21-23
18-21	70	22.3
22-25	168	53.5
26-29	40	12.7
30 and above	36	11.5
Age at menarche, years (median, IQR)	13	12-14
9-11	24	7.6
12-14	206	65.6
15 and above	84	26.8
Type of family		
Nuclear	194	61.8
Extended	81	25.8
Single parent	37	11.8
Divorced parents	2	0.6
History of pregnancy		
Yes	26	8.3
No	288	91.7
Awareness on dysmenorrhoea		
Yes	310	98.7
No	4	1.3
Regular menstrual periods		
Every time	173	55.1
Most times	91	29.0
Sometimes	34	10.8
Rarely	16	5.1

For peer review only

Duration of menstrual periods (days)		
1-2	10	3.2
3-4	95	30.3
5-6	76	24.2
7-8	19	6.1
9 and above	1	0.3

Patterns of dysmenorrhea and medication use

The majority of participants (42%, 132/314) reported experiencing menstrual cramps every time and about a quarter (23%, 71/314) reported using medicines to reduce pain during menstrual pain every time. Most (87%, 273/314) of the participants reported experiencing menstrual cramps in the past three months of whom nearly half (47%, 129/273) reported using a medication for menstrual cramps with Ibuprofen and paracetamol being the most frequently used medications (40%, 52/129 and 40%, 52/129 respectively). The majority (90%, 116/129) bought the medication from pharmacies. **Figure 1** shows the sources of the medication. **Table 2** summarizes the patterns of dysmenorrhea and medication use among female students at Makerere University.

For peer review only

Table 2. Patterns of dysmenorrhoea and medication use among female students at Makerere University

Variable	Frequency	%
Experienced menstrual pains/ cramps/ dysmenorrhea		
Every time	132	42.0
Most times	71	22.6
Sometimes	76	24.2
Rarely	35	11.2
Frequency of use of medicines (pain killers) to reduce pain during your menstrual period		
Every time	71	22.6
Most times	52	16.6
Sometimes	60	19.1
Rarely	131	41.7
Experienced menstrual cramps in the past 3 months		

Yes	273	86.9
No	41	13.1
How long ago the most recent period was (days)		
1-5	180	57.32
6-10	98	31.21
11 and above	36	11.47
Use of medication		
Yes	129	47.3
No	144	52.8
Medicines used for menstrual pains/cramps/ dysmenorrhea		
Ibuprofen	52	40.3
Paracetamol	52	40.3
Diclofenac	38	29.5
Piroxicam	26	20.2
Mefenamic Acid	9	7.0
Aspirin	6	4.7
Indomethacin	3	2.3
Dynapar	3	2.3
Brustan	3	2.3
Tramadol	2	1.6
Herb	1	0.8
Meloxicam	1	0.8

For peer review only

Prevalence of NSAID-Related GI adverse effects

Data of 129 female students who had used a medication while having dysmenorrhoea in the past three months were analysed. The prevalence of NSAID-associated GI adverse effects was 32% (n=41/129). Nausea (44%, n=18/41), ulcers (39%, n=16/41) and diarrhoea (39%, n=16/41) were the most frequently reported adverse, effects. **Figure 2**. Ibuprofen (59%, n=24/41), diclofenac (46%, n=19/41), paracetamol (15%, n=6/41), indomethacin (7%, n=3/41) and aspirin (2%, n=1/41) were implicated for the adverse effects.

Factors associated with NSAID-associated gastrointestinal adverse effects

At multivariable logistic regression, **Table 3**. Factors independently associated with GI adverse effects were: age at menarche (AOR: 1.57, 95% CI: 1.05 - 2.34), duration of menstruation (AOR: 1.57, 95% CI: 1.05 - 2.36). and use of ibuprofen (AOR: 3.87, 95% CI: 1.51 - 9.91). Females taking ibuprofen for dysmenorrhoea were about 4 times more likely to have NSAID-associated GIT adverse effects (AOR: 3.87, 95% CI: 1.51 – 9.91).

Table 3. Multivariable logistic regression showing factors associated with NSAID-associated GI adverse effects among female students at Makerere University

**Variable**	**AOR**	**95% CI**	**P**
Current age	1.00	0.74 - 1.34	0.994
Age at menarche	1.57	1.05 - 2.34	0.026
Duration of menstruation	1.57	1.05 - 2.36	0.030
Regular menstrual periods			
Every time	1.00		
Most times	1.33	0.49 - 3.59	0.574
Sometimes	0.28	0.03 - 2.76	0.275
Rarely	5.87	0.37 - 93.35	0.210
Use of NSAID for pain relief			
Every time	1.00		
Most times	0.69	0.25 - 1.90	0.470
Sometimes	0.37	0.09 - 1.61	0.187
Ibuprofen	3.87	1.51 - 9.91	0.005

Note: AOR- adjusted odds ratio, CI- confidence interval, NSAID- non steroidal anti-inflammatory disease

For peer review only

Discontinuation for NSAID-associated GI adverse effects

Overall, 68% (88/314) of the female students reported having been advised to discontinue NSAIDs (**Table 4**). Ulcers were the most common reason for discontinuation in 29 students (33%, 29/88). Of concern, some (8%, 7/88) of the students claimed that NSAIDs could cause infertility.

Table 4. Discontinuation of NSAID usage among female students at Makerere University

Variable	Frequency	%
Received advice to stop using a medication because of possible GI adverse effect		
Yes	88	68.0
No	41	32.0
Reason for advice to stop use of the NSAID?		
Ulcers	29	33.0
Infertility	7	8.0
Tolerance	3	3.4
Not good for women	2	2.3
Allergy	1	1.1
Can cause fibroids	1	1.1
Can cause cancer	1	1.1
Headache	1	1.1
Nausea	1	1.1
Menorrhagia	1	1.1
Vomiting	1	1.1
Nephrotic syndrome	1	1.1

Discussion

This study, aimed to determine the prevalence and factors associated with self-reported non-steroidal anti-inflammatory drugs (NSAIDs)-related gastrointestinal (GI) adverse effects among female students with dysmenorrhea-associated pain at Makerere University. We found a high prevalence of GI adverse effects, reaching 32%. The factors significantly associated with adverse effects included age at menarche, duration of menstruation, and ibuprofen use for pain relief.

For peer review only

Whereas there is a paucity of data on NSAID relatedGI adverse effects among female university students, previous studies conducted in India, Pakistan and Japan among patients using NSAIDs reported a prevalence of GI adverse effects, such as peptic and gastric ulcers, estimated at 30.08%, 14.7% and 16.7% respectively (18-20). In contrast to our findings, previous studies have showed that diclofenac was the commonest NSAID associated with GI adverse effects (18). The difference could be attributed to variations in the most commonly used NSAID among participants in different studies and variations in study designs. A network meta-analysis by X. Feng and X. Wang revealed that Tiaprofenic acid and mefenamic acid were indicated as the safest NSAIDs drugs, whereas indomethacin was the least safe with a higher likehood to cause mild gastrointestinal discomfort (21). In addition, a significant increase of about 2.5-5.0% in the occurrence of GI adverse effects has been observed in individuals with a history of GI incidents (22).

Comparable to findings from Ghana where the prevalence of medication use for dysmenorrhea was 46%, our study observed that 47% (129/273) of participants used medications (23). However, the frequency of the use of medication to alleviate pain greatly varied amongst the participants and could be attributed to the intensity of pain and unregulated drug use as indicated by our finding that about 90% of participants acquired the painkillers over the counter at the pharmacy (24). Contrary to a study by Moore and colleagues that showed that ibuprofen was a much safer drug than paracetamol, our study showed that females taking ibuprofen for dysmenorrhoea were about 4 times more likely to have NSAID-associated GIT adverse effects (59%) than paracetamol (15%) (25).

Whereas our study deduced that 1-year increase in the age at menarche and one-day increase in the duration of menstruation were 1.57 more likely associated with having NSAID-associated GIT adverse effects, other factors implicated in other studies include; *Helicobacter pylori* infection,

For peer review only

high dose or multiple NSAID use, history of upper GI injury, receiving hemodialysis and anticoagulant, oral corticosteroid or selective serotonin reuptake inhibitor use (26-29).

GI adverse effects are associated with increased physician visits, risk of GI surgery, high health care costs and poorer health related quality of life (HRQoL) (30). However, potential strategies such as the use of GI sparing NSAIDs, use of muco-protective drugs and alternative medications or no pharmacological remedies for pain relief have been adopted in order to prevent NSAID induced GI adverse effects (31, 32).

Our study contributes to the existing literature on the current status of NSAID-related GI effects among female university students in Uganda. However, there were some limitations. Due to the COVID-19 pandemic, the study was conducted online. Therefore, a limited number of students with internet access participated. This also limited the nature of study participant selection as we were unable to use probability sampling methods, the sampling technique used may not have given us the best representative study population. In addition, the exclusive use of a quantitative design in our study prevented us from exploring important perspectives that could have been derived from a qualitative study. While we performed statistical analyses to examine variable associations, we did not explicitly test for potential confounding and interaction effects, which may exist and have the potential to influence our findings.

Conclusion

In this study, we found a considerably high prevalence of NSAID-related GI adverse effects associated with; age at menarche, longer duration of menstruation, and ibuprofen use among female students at Makerere University experienced dysmenorrhea. Therefore, efforts directed towards mass education of females about the prevention, early identification and management of

For peer review only

NSAID-related GI adverse effects are necessary. We recommend further exploration of alternative strategies for pain relief for females with dysmenorrhea.

Acknowledgments

The authors acknowledge the contribution of the following team of individuals in the data collection process. The team is as follows; Dr. Haruna Kiryowa, Mr. David Bakka, Mr. Gerald Akena, Mr. Samuel Ssenkungu, Ms. Catherine Aiya Lalam and Ms. Gillian Sheila Amuge,

Contributorship statement

All authors contributed significantly to study conceptualisation, data collection, analysis, interpretation, and manuscript writing.

GS, KW and RK contributed to the study concept and design, and developed the questionnaire. GS and KW recruited collaborators for survey distribution and data collection. GS, KR and KW contributed to data acquisition, analysis and interpretation. KW wrote the initial draft and all authors participated in subsequent writing and critical revision of the manuscript.

RK oversaw the project implementation. KW managed project administration.

All authors approved the final version to be published and are accountable for all aspects of the work.

Competing interests

All authors declare that they have no conflict of interest.

Funding

This research was financially supported by Medical Research Council (MR/V03510X/1).

For peer review only

Data sharing statement

No additional data are available.

Figure Captions

Figure 1. Sources of medication for menstrual cramps relief among female students at Makerere University

Figure 2. Frequency of NSAID-associated GI adverse effects among female students at Makerere University

References

1. Kumar KH, Elavarasi PJJoAC, Insights R. Definition of pain and classification of pain disorders. 2016;3(3):87-90.
2. Smith RP. The clinical classification and causes of dysmenorrhea. Dysmenorrhea and Menorrhagia: Springer; 2018. p. 55-64.
3. Parry K, Armour M, Smith CA, MacMillan F, Manohar N, Holmes K, et al. The Prevalence and Academic Impact of Dysmenorrhea in 21,573 Young Women: A Systematic Review and Meta-Analysis. Journal of Women's Health (15409996). 2019;28(8).
4. Dawood MY. Primary dysmenorrhea: advances in pathogenesis and management. Obstetrics & Gynecology. 2006;108(2):428-41.
5. Proctor MML, Farquhar CM. Dysmenorrhoea. BMJ clinical evidence. 2007;2007.
6. Vincenzo De Sanctis M, Soliman A, Bernasconi S, Bianchin L, Bona G, Bozzola M, et al. Primary dysmenorrhea in adolescents: prevalence, impact and recent knowledge. 2015;13(2):465-73.
7. Unsal A, Ayranci U, Tozun M, Arslan G, Calik EJUjoms. Prevalence of dysmenorrhea and its effect on quality of life among a group of female university students. 2010;115(2):138-45.
8. Ameade EPK, Amalba A, Mohammed BSJBWsH. Prevalence of dysmenorrhea among University students in Northern Ghana; its impact and management strategies. 2018;18(1):1-9.
9. Nooh AMJMEFSJ. Menstrual disorders among Zagazig university students, Zagazig, Egypt. 2015;20(3):198-203.
10. Nakame RM, Kiwanuka F, Robert AJNo. Dysmenorrhoea among students aged 18–45 years attending University in Uganda: A cross-sectional multicenter study of three Universities in Uganda. 2019;6(2):268-75.
11. Alshakka MA, Badullah WF, Alolayan SO, Mahmoud MAJBR. Prescribing patterns of non-steroidal anti-inflammatory drugs (NSAIDs) at outpatient departments of four hospitals. 2018;29(19):3643-7.

For peer review only

12. Duong M, Salvo F, Pariente A, Abouelfath A, Lassalle R, Droz C, et al. Usage patterns of 'over-the-counter'vs. prescription-strength nonsteroidal anti-inflammatory drugs in F rance. 2014;77(5):887-95.
13. Wilcox CM, Cryer B, Triadafilopoulos GJTJor. Patterns of use and public perception of over-the-counter pain relievers: focus on nonsteroidal antiinflammatory drugs. 2005;32(11):2218-24.
14. Castellsague J, Riera-Guardia N, Calingaert B, Varas-Lorenzo C, Fourrier-Reglat A, Nicotra F, et al. Individual NSAIDs and upper gastrointestinal complications. Drug safety. 2012;35(12):1127-46.
15. Vostinaru O. Adverse Effects and Drug Interactions of the Non-Steroidal Anti-Inflammatory Drugs. Nonsteroidal Anti-Inflammatory Drugs. 2017:17.
16. MacDonald T. Epidemiology and pharmacoeconomic implications of non-steroidal anti-inflammatory drug-associated gastrointestinal toxicity. Rheumatology. 2000;39(suppl_2):13-20.
17. Kish L. Sampling organizations and groups of unequal sizes. American sociological review. 1965:564-72.
18. Hamid S, Yakoob J, Jafri W, Islam S, Abid S, Islam MJJoPMA. Frequency of NSAID induced peptic ulcer disease. 2006;56(5):218.
19. Chatterjee S, Dureja GP, Kadhe G, Mane A, Phansalkar AA, Sawant S, et al. Cross-sectional study for prevalence of non-steroidal anti-inflammatory drug-induced gastrointestinal, cardiac and renal complications in India: interim report. 2015;8(3-4):216.
20. Tsujimoto S, Mokuda S, Matoba K, Yamada A, Jouyama K, Murata Y, et al. The prevalence of endoscopic gastric mucosal damage in patients with rheumatoid arthritis. 2018;13(7):e0200023.
21. Feng X, Wang X. Comparison of the efficacy and safety of non-steroidal anti-inflammatory drugs for patients with primary dysmenorrhea: A network meta-analysis. Molecular Pain. 2018;14:1744806918770320.
22. Dubois R, Melmed G, Henning J, Laine LJAp, therapeutics. Guidelines for the appropriate use of non-steroidal anti-inflammatory drugs, cyclo-oxygenase-2-specific inhibitors and proton pump inhibitors in patients requiring chronic anti-inflammatory therapy. 2004;19(2):197-208.
23. Ameade EPK, Amalba A, Mohammed BS. Prevalence of dysmenorrhea among University students in Northern Ghana; its impact and management strategies. BMC women's health. 2018;18(1):39.
24. Samba Conney C, Akwo Kretchy I, Asiedu-Danso M, Allotey-Babington GLJO, International G. Complementary and alternative medicine use for primary dysmenorrhea among senior high school students in the western region of Ghana. 2019;2019.
25. Moore NJIjocpS. Forty years of ibuprofen use. 2003(135):28-31.
26. Goldstein JL, Cryer BJD, healthcare, safety p. Gastrointestinal injury associated with NSAID use: a case study and review of risk factors and preventative strategies. 2015;7:31.
27. Jankovic SM, Aleksic J, Rakovic S, Aleksic A, Stevanovic I, Stefanovic-Stoimenov N, et al. Nonsteroidal antiinflammatory drugs and risk of gastrointestinal bleeding among patients on hemodialysis. 2009;22(4):502.
28. García Rodríguez LA, Hernández-Díaz SJAR, Therapy. The risk of upper gastrointestinal complications associated with nonsteroidal anti-inflammatory drugs, glucocorticoids, acetaminophen, and combinations of these agents. 2001;3(2):1-4.

For peer review only

29. Shorr RI, Ray WA, Daugherty JR, Griffin MRJAoim. Concurrent use of nonsteroidal anti-inflammatory drugs and oral anticoagulants places elderly persons at high risk for hemorrhagic peptic ulcer disease. 1993;153(14):1665-70.
30. Chang LJAp, therapeutics. Epidemiology and quality of life in functional gastrointestinal disorders. 2004;20:31-9.
31. Guo C-G, Leung WKJG, liver. Potential strategies in the prevention of nonsteroidal anti-inflammatory drugs-associated adverse effects in the lower gastrointestinal tract. 2020;14(2):179.
32. García-Rayado G, Navarro M, Lanas AJErocp. NSAID induced gastrointestinal damage and designing GI-sparing NSAIDs. 2018;11(10):1031-43.

For peer review only

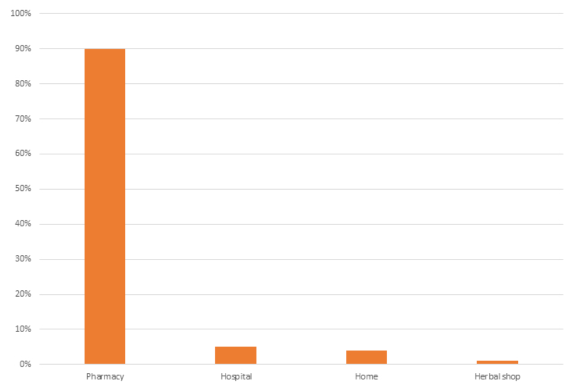

338x190mm (96 x 96 DPI)

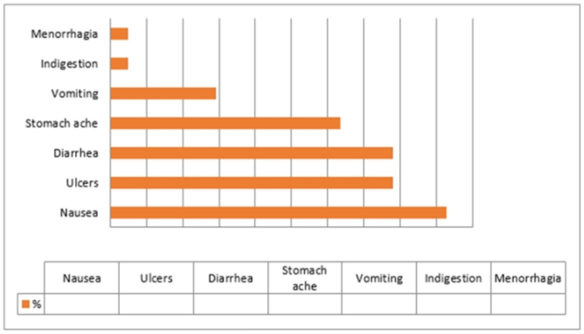

338x190mm (96 x 96 DPI)

STROBE Statement—Checklist of items that should be included in reports of ***cross-sectional studies***

	Item No	Recommendation
**Title and abstract**	1	(*a*) Indicate the study's design with a commonly used term in the title or the abstract-Page 1
		(*b*) Provide in the abstract an informative and balanced summary of what was done and what was found-Page 2
**Introduction**		
Background/rationale	2	Explain the scientific background and rationale for the investigation being reported-Page 3, 4
Objectives	3	State specific objectives, including any prespecified hypotheses-Page 4
**Methods**		
Study design	4	Present key elements of study design early in the paper-Page 4
Setting	5	Describe the setting, locations, and relevant dates, including periods of recruitment, exposure, follow-up, and data collection-Page 4,5
Participants	6	(*a*) Give the eligibility criteria, and the sources and methods of selection of participants-Page 5
Variables	7	Clearly define all outcomes, exposures, predictors, potential confounders, and effect modifiers. Give diagnostic criteria, if applicable-Page 6
Data sources/ measurement	8*	For each variable of interest, give sources of data and details of methods of assessment (measurement). Describe comparability of assessment methods if there is more than one group-Page 5
Bias	9	Describe any efforts to address potential sources of bias-Page 5
Study size	10	Explain how the study size was arrived at- page 5
Quantitative variables	11	Explain how quantitative variables were handled in the analyses. If applicable, describe which groupings were chosen and why-Page 6
Statistical methods	12	(*a*) Describe all statistical methods, including those used to control for confounding-Page 7
		(*b*) Describe any methods used to examine subgroups and interactions-Page 7
		(*c*) Explain how missing data were addressed-Page 7
		(*d*) If applicable, describe analytical methods taking account of sampling strategy-Page 7
		(*e*) Describe any sensitivity analyses-Page 7
**Results**		
Participants	13*	(a) Report numbers of individuals at each stage of study—eg numbers potentially eligible, examined for eligibility, confirmed eligible, included in the study, completing follow-up, and analysed-Page 7
Descriptive data	14*	(a) Give characteristics of study participants (eg demographic, clinical, social) and information on exposures and potential confounders-Page 7,8
		(b) Indicate number of participants with missing data for each variable of interest-Page 7
Outcome data	15*	Report numbers of outcome events or summary measures-Page 9
Main results	16	(*a*) Give unadjusted estimates and, if applicable, confounder-adjusted estimates and their precision (eg, 95% confidence interval). Make clear which confounders were adjusted for and why they were included-Page 12, 13

For peer review only - http://bmjopen.bmj.com/site/about/guidelines.xhtml

Other analyses	17	Report other analyses done—eg analyses of subgroups and interactions, and sensitivity analyses-Page 13
**Discussion**		
Key results	18	Summarise key results with reference to study objectives-page 14
Limitations	19	Discuss limitations of the study, taking into account sources of potential bias or imprecision. Discuss both direction and magnitude of any potential bias-Page 15
Interpretation	20	Give a cautious overall interpretation of results considering objectives, limitations, multiplicity of analyses, results from similar studies, and other relevant evidence-Page 14, 15
Generalisability	21	Discuss the generalisability (external validity) of the study results-Page 14, 15
**Other information**		
Funding	22	Give the source of funding and the role of the funders for the present study and, if applicable, for the original study on which the present article is based-Page 16

For peer review only

BMJ Open

Self-reported gastrointestinal adverse effects of non-steroidal anti-inflammatory drugs in female students with dysmenorrhea at Makerere University: Prevalence, discontinuation and associated factors: A cross sectional study

Journal:	*BMJ Open*
Manuscript ID	bmjopen-2023-079660.R2
Article Type:	Original research
Date Submitted by the Author:	11-Apr-2024
Complete List of Authors:	Gobba, Solomon; Makerere University<br>Kibone, Winnie; Makerere University<br>Kiguba, Ronald; Makerere University College of Health Sciences, Pharmacology and Therapeutics
**Primary Subject Heading**:	Pharmacology and therapeutics
Secondary Subject Heading:	Reproductive medicine
Keywords:	Dysmenorrhea, Adverse events < THERAPEUTICS, REPRODUCTIVE MEDICINE

SCHOLARONE™
Manuscripts

I, the Submitting Author has the right to grant and does grant on behalf of all authors of the Work (as defined in the below author licence), an exclusive licence and/or a non-exclusive licence for contributions from authors who are: i) UK Crown employees; ii) where BMJ has agreed a CC-BY licence shall apply, and/or iii) in accordance with the terms applicable for US Federal Government officers or employees acting as part of their official duties; on a worldwide, perpetual, irrevocable, royalty-free basis to BMJ Publishing Group Ltd ("BMJ") its licensees and where the relevant Journal is co-owned by BMJ to the co-owners of the Journal, to publish the Work in this journal and any other BMJ products and to exploit all rights, as set out in our licence.

The Submitting Author accepts and understands that any supply made under these terms is made by BMJ to the Submitting Author unless you are acting as an employee on behalf of your employer or a postgraduate student of an affiliated institution which is paying any applicable article publishing charge ("APC") for Open Access articles. Where the Submitting Author wishes to make the Work available on an Open Access basis (and intends to pay the relevant APC), the terms of reuse of such Open Access shall be governed by a Creative Commons licence – details of these licences and which Creative Commons licence will apply to this Work are set out in our licence referred to above.

Other than as permitted in any relevant BMJ Author's Self Archiving Policies, I confirm this Work has not been accepted for publication elsewhere, is not being considered for publication elsewhere and does not duplicate material already published. I confirm all authors consent to publication of this Work and authorise the granting of this licence.

For peer review only

Self-reported gastrointestinal adverse effects of non-steroidal anti-inflammatory drugs in female students with dysmenorrhea at Makerere University: Prevalence, discontinuation and associated factors: A cross sectional study

Solomon Gobba[1], Winnie Kibone[2], Ronald Kiguba[3]

Affiliations

[1]School of Health Sciences, College of Health Sciences, Makerere University, Kampala, Uganda

[2]School of Medicine, College of Health Sciences, Makerere University, Kampala, Uganda

[3] School of Biomedical Sciences, College of Health Sciences, Makerere University, Kampala, Uganda

***Corresponding author**

Gobba Solomon
School of Health Sciences, College of Health Sciences, Makerere University, Kampala, Uganda
Email: solomongobba@gmail.com

Author details

Solomon Gobba: solomongobba@gmail.com
Winnie Kibone: kibonewinnie@gmail.com
Ronald Kiguba: kiguba@gmail.com

For peer review only

Abstract

Background: Primary dysmenorrhea occurs in up to 50% of menstruating females. Non-Steroidal Anti-inflammatory Drugs (NSAIDS) are the most commonly used therapeutic remedies for dysmenorrhea in Uganda. However, NSAIDS are associated with a 3-to-5-fold increase in the risk of gastrointestinal (GI) adverse drug effects. We aimed to determine the prevalence and associated factors of self-reported NSAID-related GI adverse effects in female students who use NSAIDs in managing dysmenorrhea-associated pain at Makerere University.

Method: We conducted a cross-sectional study among female students at Makerere University, Kampala, Uganda from 14th October 2020 to 29th October 2020. Socio-demographic, menstrual history and treatment data were collected online using a pretested questionnaire, cleaned using Microsoft Excel 2016 and analyzed with STATA 15.1 software. Logistic regression was used to determine factors associated with self-reported adverse effects of NSAIDs among the female students. $p < 0.05$ was considered statistically significant.

Results: Overall, 314 valid responses were received from female students with a median age of 22 years (IQR: 18 – 29 years). The median age at menarche was 13 years (IQR: 9 – 18 years). 41% (n=129/314) of the respondents had used medication for dysmenorrhea, and 32% (n=41/129) of whom reported an NSAID-associated GI adverse effects with nausea being the most frequently reported (44%, n=18/41)

Factors independently associated with GI adverse effects were: age at menarche (p=0.026), duration of menstruation (P=0.030) and use of ibuprofen (p=0.005). Females taking ibuprofen for dysmenorrhoea were about 4 times as likely to have NSAID-associated GI adverse effects (AOR: 3.87, 95% CI: 1.51 – 9.91) than those who didn't receive ibuprofen.

Conclusion: We found a considerably high prevalence of NSAID-related GI adverse effects driven by factors such as age at menarche and ibuprofen use.

For peer review only

Keywords: NSAIDs, gastrointestinal (GI), adverse effect, dysmenorrhea, Uganda.

Strengths and Limitations of the study

Only a limited number of students with internet access participated in the study as it was conducted online during the COVID-19 pandemic.

The response rate could not be determined since it was hard to accurately ascertain the number of participants reached

sampling methods that encourage randomisation could not be employed in participant selection.

Introduction

Dysmenorrhea is defined as painful menstrual cramps and is classified into primary dysmenorrhea without any evident pathology and secondary dysmenorrhea with a pathology (1, 2). Pinpointing the exact prevalence remains elusive, as studies conducted in diverse populations show varying rates, spanning from 20% to 94% (3) Primary dysmenorrhea occurs in up to 50% of menstruating females and causes significant disruption in quality of life and absenteeism from school and work places affecting their productivity (4). The pain is attributed to uterine prostaglandins released by endometrial cells during uterine wall sloughing prior to the onset of menstruation (5). On account of the high prevalence of dysmenorrhea with a global prevalence ranging between 20% to 90%, dysmenorrhea is a serious public health burden (6). Studies have documented an equally higher prevalence of dysmenorrhea estimated at about 65.4%, 72.7% and 83.6% in Egypt, Ghana and Turkey respectively (7-9). In Uganda, the prevalence of dysmenorrhea has been estimated at 75.8% (10).

Non-steroidal anti-inflammatory drugs (NSAIDs) remain the most commonly used therapeutic remedies for dysmenorrhea in Uganda because of their affordability, convenience of use and

For peer review only

efficacy (11). NSAIDs are commonly used as over-the-counter drugs and may often not be prescribed (12, 13). NSAIDs relieve symptoms in up to 70% of women when used correctly. Furthermore, NSAIDs are the recommended first-line treatment in management of dysmenorrhea unless there are contraindications such as history of hypersensitivity to aspirin or other NSAIDs, serious comorbidity, and GI ulcers or bleeding (5).

The utilization of NSAIDs has been linked to a significant 3- to 5-fold increase in the risk of GI adverse effects (14). Dyspepsia with pyrosis, abdominal pain, nausea, anorexia, gastric erosions, ulcers, perforation, GI hemorrhage which may result in anemia are some of the commonly observed GI adverse effects in NSAID therapy (15). Studies have explored GI complications of NSAID-related treatment in cases of rheumatoid arthritis and osteoarthritis in the elderly (16) but little is known in relation to GI adverse drug effects related to NSAIDs therapy in the management of dysmenorrhea. Furthermore, university students practice more self-medication using NSAIDs for dysmenorrhea. In fact, recent publication showed a high prevalence of self-medication estimated at 37% commonly mefenamic acid (58%).

This study, aimed to determine the commonly used NSAIDs, the prevalence of self-reported NSAID-related GI adverse effects and the factors associated with self-reported NSAID-related GI adverse effects during the management of dysmenorrhea among female students at Makerere University, Kampala, Uganda.

Methods

Study design

A descriptive cross-sectional study was conducted, between 14th October 2020 and 29th October 2020 to determine the prevalence and factors associated with GI adverse effects to NSAIDs usage in the management of dysmenorrhea among female students at Makerere University.

For peer review only

Study setting

The study was conducted at Makerere University main campus, situated North of Kampala, the capital city of Uganda, at the coordinates 0.3293° N, 32.5711° E. Makerere University is the largest public university in Uganda, attracting students from various regions of the country. The university accommodates over 40,000 students, both resident and non-resident. The main campus features twelve Halls of Residence, with six for male students and three for female students. Medical students are accommodated at the Mulago Hospital Complex. Approximately 5,000 resident undergraduates and 100 graduate students, accounting for 13% of the university's registered students, reside in the Halls of Residence. The remaining students either live in private hostels or commute from their homes. The age range of the majority of students falls between 20 and 30 years, encompassing a diverse representation of socio-economic statuses.

Study population

Female students pursuing an undergraduate program at Makerere University and residing in different halls of residence and hostels. The sample size of 314 participants was calculated using the modified Kish and Leslie formula(17) for finite population size, with an estimated prevalence of dysmenorrhea among university students (75.8%) , and a 10% adjustment was made to cater for nonresponse.

Selection criteria

All registered female students of Makerere University aged 18 years and older were eligible for the study after informed consent. Purposive sampling technique was used to select students into the study. Female students with primary or secondary amenorrhea, in menopause, with mental incapacitation or without access to internet were excluded from the study.

For peer review only

Data Collection

We used a self-administered questionnaire to collect data for this study. The questionnaire was pre-tested for comprehension and appropriateness among 15 students and adjusted accordingly to ensure quality data collection. The data were collected online via a Google survey document where participants received the survey information via a link shared with them in their WhatsApp groups and in their personal email inboxes. Eligible participants consented to the study and completed the survey. The submitted survey responses were automatically stored in an online data bank, accessible only to the Principal Investigator (PI) and the Supervisor. Additionally, the data were backed up on the Principal Investigator's Google Drive to ensure data security. The questionnaire variables were constituted in three parts: sociodemographic information, menstrual history, and treatment variables. These sections aimed to gather comprehensive data relevant to the study objectives.

Study Variables

Independent variables: social demographics (age, and history of pregnancy), menstrual history (dysmenorrhea, menarche, regularity and duration of menstrual periods) and treatment (painkillers used and their source, side effects).

Dependent variables: NSAID-related GI adverse effects.

Data quality control

To ensure that high quality data were captured from the volunteers, the data collection tool was pretested and adjusted to ensure convenience and ease of manoeuvre. Checks were incorporated within the document to ensure all questions were responded to and correctly answered to reduce the margins of error.

For peer review only

Data management

Data collection tools were checked for completeness and instantly submitted to a central data bank that tallied all responses received from the participants. The central databank was synchronized and backed up on the PI's google drive for secondary storage. Upon completion of the survey, the data were translated into Excel for data cleaning and initial analysis. The data were then exported to STATA Software (StataCorp LLC, USA) for further analysis.

Data analysis

All analyses were performed using Microsoft Excel 2016 and STATA Software. Numerical data were summarized as means and standard deviations if normally distributed; otherwise, median and interquartile ranges were used for non-normally distributed data. Categorical data were summarized as frequencies and proportions with their 95% confidence intervals. Associations between independent variables and dependent variables were assessed using the chi-square test and multivariable analysis using STATA 15.1 software. A P-Value of less than 0.05 was considered statistically significant.

Ethical and Regulatory Approval

We obtained ethical approval from of the School of Biomedical Sciences Research and Ethics Committee, Makerere University prior to study implementation, REC Approval Number SBS-REC -888. Participation in the study was voluntary and participants provided informed consent prior to enrolment into the study. All information was kept confidential and used for research purposes only.

Patient and Public Involvement

For peer review only

Patients and the public were at the centre of this research, our research questions and outcome measures were shaped by findings from previous studies which often reflect patient-reported outcomes, adverse events, and medication-related errors. We aimed to develop research objectives that were meaningful, relevant, and actionable in addressing medication safety issues from a patient-centered standpoint.

We conducted a questionnaire pretest session with a select group of participants to refine study questionnaire which ensured that the study design was sensitive to patient preferences and experiences.

The results of the study will be disseminated to study participants and the general public through various channels to ensure accessibility and comprehension. This will include the distribution of lay summaries written in plain language, presentations at patient-focused conferences, and publication of study findings in open-access journals.

For peer review only

Results

Characteristics of Participants

Overall, a total of 314 participants responded to the study. The median age was 22 years (IQR: 21-23). The majority came from nuclear families (62%, 194/314). Only 8% (26/314) of the participants had a history of pregnancy. The median age at menarche was 13 years (IQR: 12-14) with range of 9 to 18 years and more than half (55%, 173/314) experienced regular periods every

time. Nearly all female students were aware of dysmenorrhoea (99%, 310/314). **Table 1** summarizes the baseline characteristics of participants.

Table 1. Demographic and Menstrual Characteristics of Participants

Variable	Frequency (n)	Percentage (%)
Age of respondents, years (Median, IQR)	22	21-23
18-21	70	22.3
22-25	168	53.5
26-29	40	12.7
30 and above	36	11.5
Age at menarche, years (median, IQR)	13	12-14
9-11	24	7.6
12-14	206	65.6
15 and above	84	26.8
Type of family		
Nuclear	194	61.8
Extended	81	25.8
Single parent	37	11.8
Divorced parents	2	0.6
History of pregnancy		
Yes	26	8.3
No	288	91.7
Awareness on dysmenorrhoea		
Yes	310	98.7
No	4	1.3
Regular menstrual periods		
Every time	173	55.1
Most times	91	29.0
Sometimes	34	10.8
Rarely	16	5.1
Duration of menstrual periods (days)		
1-2	10	3.2
3-4	95	30.3
5-6	76	24.2
7-8	19	6.1
9 and above	1	0.3

For peer review only

For peer review only - http://bmjopen.bmj.com/site/about/guidelines.xhtml

Patterns of dysmenorrhea and medication use

The majority of participants (42%, 132/314) reported experiencing menstrual cramps every time and about a quarter (23%, 71/314) reported using medicines to reduce pain during menstrual pain every time. Most (87%, 273/314) of the participants reported experiencing menstrual cramps in the past three months of whom nearly half (47%, 129/273) reported using a medication for menstrual cramps with Ibuprofen and paracetamol being the most frequently used medications (40%, 52/129 and 40%, 52/129 respectively). The majority (90%, 116/129) bought the medication from pharmacies. **Figure 1** shows the sources of the medication. **Table 2** summarizes the patterns of dysmenorrhea and medication use among female students at Makerere University.

Table 2. Patterns of dysmenorrhoea and medication use among female students at Makerere University

Variable	Frequency	%
Experienced menstrual pains/ cramps/ dysmenorrhea		
Every time	132	42.0
Most times	71	22.6
Sometimes	76	24.2
Rarely	35	11.2
Frequency of use of medicines (pain killers) to reduce pain during your menstrual period		
Every time	71	22.6
Most times	52	16.6
Sometimes	60	19.1
Rarely	131	41.7
Experienced menstrual cramps in the past 3 months		
Yes	273	86.9
No	41	13.1
How long ago the most recent period was (days)		
1-5	180	57.32
6-10	98	31.21
11 and above	36	11.47
Use of medication		
Yes	129	47.3
No	144	52.8

For peer review only

Medicines used for menstrual pains/cramps/ dysmenorrhea		
Ibuprofen	52	40.3
Paracetamol	52	40.3
Diclofenac	38	29.5
Piroxicam	26	20.2
Mefenamic Acid	9	7.0
Aspirin	6	4.7
Indomethacin	3	2.3
Dynapar	3	2.3
Brustan	3	2.3
Tramadol	2	1.6
Herb	1	0.8
Meloxicam	1	0.8

Prevalence of NSAID-Related GI adverse effects

Data of 129 female students who had used a medication while having dysmenorrhoea in the past three months were analysed. The prevalence of NSAID-associated GI adverse effects was 32% (n=41/129). Nausea (44%, n=18/41), ulcers (39%, n=16/41) and diarrhoea (39%, n=16/41) were the most frequently reported adverse, effects. **Figure 2**. Ibuprofen (59%, n=24/41), diclofenac (46%, n=19/41), paracetamol (15%, n=6/41), indomethacin (7%, n=3/41) and aspirin (2%, n=1/41) were implicated for the adverse effects.

Factors associated with NSAID-associated gastrointestinal adverse effects

At multivariable logistic regression, **Table 3**. Factors independently associated with GI adverse effects were: age at menarche (AOR: 1.57, 95% CI: 1.05 - 2.34), duration of menstruation (AOR: 1.57, 95% CI: 1.05 - 2.36). and use of ibuprofen (AOR: 3.87, 95% CI: 1.51 - 9.91). Females taking

For peer review only

ibuprofen for dysmenorrhoea were about 4 times more likely to have NSAID-associated GIT adverse effects (AOR: 3.87, 95% CI: 1.51 – 9.91).

Table 3. Multivariable logistic regression showing factors associated with NSAID-associated GI adverse effects among female students at Makerere University

Variable	AOR	95% CI	P
Current age	1.00	0.74 - 1.34	0.994
Age at menarche	1.57	1.05 - 2.34	0.026
Duration of menstruation	1.57	1.05 - 2.36	0.030
Regular menstrual periods			
Every time	1.00		
Most times	1.33	0.49 - 3.59	0.574
Sometimes	0.28	0.03 - 2.76	0.275
Rarely	5.87	0.37 - 93.35	0.210
Use of NSAID for pain relief			
Every time	1.00		
Most times	0.69	0.25 - 1.90	0.470
Sometimes	0.37	0.09 - 1.61	0.187
Ibuprofen	3.87	1.51 - 9.91	0.005

Note: AOR- adjusted odds ratio, CI- confidence interval, NSAID- non steroidal anti-inflammatory disease

For peer review only

Discontinuation for NSAID-associated GI adverse effects

Overall, 68% (88/314) of the female students reported having been advised to discontinue NSAIDs (**Table 4**). Ulcers were the most common reason for discontinuation in 29 students (33%, 29/88). Of concern, some (8%, 7/88) of the students claimed that NSAIDs could cause infertility.

Table 4. Discontinuation of NSAID usage among female students at Makerere University

Variable	Frequency	%
Received advice to stop using a medication because of possible GI adverse effect		
Yes	88	68.0
No	41	32.0
Reason for advice to stop use of the NSAID?		
Ulcers	29	33.0
Infertility	7	8.0
Tolerance	3	3.4
Not good for women	2	2.3
Allergy	1	1.1
Can cause fibroids	1	1.1
Can cause cancer	1	1.1
Headache	1	1.1
Nausea	1	1.1
Menorrhagia	1	1.1
Vomiting	1	1.1
Nephrotic syndrome	1	1.1

For peer review only

Discussion

This study, aimed to determine the prevalence and factors associated with self-reported non-steroidal anti-inflammatory drugs (NSAIDs)-related gastrointestinal (GI) adverse effects among female students with dysmenorrhea-associated pain at Makerere University. We found a high prevalence of GI adverse effects, reaching 32%. The factors significantly associated with adverse effects included age at menarche, duration of menstruation, and ibuprofen use for pain relief.

Whereas there is a paucity of data on NSAID relatedGI adverse effects among female university students, previous studies conducted in India, Pakistan and Japan among patients using NSAIDs reported a prevalence of GI adverse effects, such as peptic and gastric ulcers, estimated at 30.08%, 14.7% and 16.7% respectively (18-20). In contrast to our findings, previous studies have showed that diclofenac was the commonest NSAID associated with GI adverse effects (18). The difference could be attributed to variations in the most commonly used NSAID among participants in

different studies and variations in study designs. A network meta-analysis by X. Feng and X. Wang revealed that Tiaprofenic acid and mefenamic acid were indicated as the safest NSAIDs drugs, whereas indomethacin was the least safe with a higher likehood to cause mild gastrointestinal discomfort (21). In addition, a significant increase of about 2.5-5.0% in the occurrence of GI adverse effects has been observed in individuals with a history of GI incidents (22).

Comparable to findings from Ghana where the prevalence of medication use for dysmenorrhea was 46%, our study observed that 47% (129/273) of participants used medications (23). However, the frequency of the use of medication to alleviate pain greatly varied amongst the participants and could be attributed to the intensity of pain and unregulated drug use as indicated by our finding that about 90% of participants acquired the painkillers over the counter at the pharmacy (24). Contrary to a study by Moore and colleagues that showed that ibuprofen was a much safer drug than paracetamol, our study showed that females taking ibuprofen for dysmenorrhoea were about 4 times more likely to have NSAID-associated GIT adverse effects (59%) than paracetamol (15%) (25).

Whereas our study deduced that 1-year increase in the age at menarche and one-day increase in the duration of menstruation were 1.57 more likely associated with having NSAID-associated GIT adverse effects, other factors implicated in other studies include; *Helicobacter pylori* infection, high dose or multiple NSAID use, history of upper GI injury, receiving hemodialysis and anticoagulant, oral corticosteroid or selective serotonin reuptake inhibitor use (26-29).

GI adverse effects are associated with increased physician visits, risk of GI surgery, high health care costs and poorer health related quality of life (HRQoL) (30). However, potential strategies such as the use of GI sparing NSAIDs, use of muco-protective drugs and alternative medications

For peer review only

275 or no pharmacological remedies for pain relief have been adopted in order to prevent NSAID
276 induced GI adverse effects (31, 32).

277 Our study contributes to the existing literature on the current status of NSAID-related GI effects
278 among female university students in Uganda. However, there were some limitations. Due to the
279 COVID-19 pandemic, the study was conducted online. Therefore, a limited number of students
280 with internet access participated. This also limited the nature of study participant selection as we
281 were unable to use probability sampling methods, the sampling technique used may not have given
282 us the best representative study population. In addition, the exclusive use of a quantitative design
283 in our study prevented us from exploring important perspectives that could have been derived from
284 a qualitative study. While we performed statistical analyses to examine variable associations, we
285 did not explicitly test for potential confounding and interaction effects, which may exist and have
286 the potential to influence our findings.

287 Conclusion

288 In this study, we found a considerably high prevalence of NSAID-related GI adverse effects
289 associated with; age at menarche, longer duration of menstruation, and ibuprofen use among
290 female students at Makerere University experienced dysmenorrhea. Therefore, efforts directed
291 towards mass education of females about the prevention, early identification and management of
292 NSAID-related GI adverse effects are necessary. We recommend further exploration of alternative
293 strategies for pain relief for females with dysmenorrhea.

294 Acknowledgments

For peer review only

The authors acknowledge the contribution of the following team of individuals in the data collection process. The team is as follows; Dr. Haruna Kiryowa, Mr. David Bakka, Mr. Gerald Akena, Mr. Samuel Ssenkungu, Ms. Catherine Aiya Lalam and Ms. Gillian Sheila Amuge,

Contributorship statement

All authors contributed significantly to study conceptualisation, data collection, analysis, interpretation, and manuscript writing.

GS, KW and RK contributed to the study concept and design, and developed the questionnaire. GS and KW recruited collaborators for survey distribution and data collection. GS, KR and KW contributed to data acquisition, analysis and interpretation. KW wrote the initial draft and all authors participated in subsequent writing and critical revision of the manuscript.

RK oversaw the project implementation. KW managed project administration.

All authors approved the final version to be published and are accountable for all aspects of the work.

Competing interests

All authors declare that they have no conflict of interest.

Funding

This research was financially supported by Medical Research Council (MR/V03510X/1).

Data sharing statement

No additional data are available.

Figure Captions

For peer review only

Figure 1. Sources of medication for menstrual cramps relief among female students at Makerere University

Figure 2. Frequency of NSAID-associated GI adverse effects among female students at Makerere University

References

1. Kumar KH, Elavarasi PJJoAC, Insights R. Definition of pain and classification of pain disorders. 2016;3(3):87-90.
2. Smith RP. The clinical classification and causes of dysmenorrhea. Dysmenorrhea and Menorrhagia: Springer; 2018. p. 55-64.
3. Parry K, Armour M, Smith CA, MacMillan F, Manohar N, Holmes K, et al. The Prevalence and Academic Impact of Dysmenorrhea in 21,573 Young Women: A Systematic Review and Meta-Analysis. Journal of Women's Health (15409996). 2019;28(8).
4. Dawood MY. Primary dysmenorrhea: advances in pathogenesis and management. Obstetrics & Gynecology. 2006;108(2):428-41.
5. Proctor MML, Farquhar CM. Dysmenorrhoea. BMJ clinical evidence. 2007;2007.
6. Vincenzo De Sanctis M, Soliman A, Bernasconi S, Bianchin L, Bona G, Bozzola M, et al. Primary dysmenorrhea in adolescents: prevalence, impact and recent knowledge. 2015;13(2):465-73.
7. Unsal A, Ayranci U, Tozun M, Arslan G, Calik EJUjoms. Prevalence of dysmenorrhea and its effect on quality of life among a group of female university students. 2010;115(2):138-45.
8. Ameade EPK, Amalba A, Mohammed BSJBWsH. Prevalence of dysmenorrhea among University students in Northern Ghana; its impact and management strategies. 2018;18(1):1-9.
9. Nooh AMJMEFSJ. Menstrual disorders among Zagazig university students, Zagazig, Egypt. 2015;20(3):198-203.
10. Nakame RM, Kiwanuka F, Robert AJNo. Dysmenorrhoea among students aged 18–45 years attending University in Uganda: A cross-sectional multicenter study of three Universities in Uganda. 2019;6(2):268-75.
11. Alshakka MA, Badullah WF, Alolayan SO, Mahmoud MAJBR. Prescribing patterns of non-steroidal anti-inflammatory drugs (NSAIDs) at outpatient departments of four hospitals. 2018;29(19):3643-7.
12. Duong M, Salvo F, Pariente A, Abouelfath A, Lassalle R, Droz C, et al. Usage patterns of ‘over-the-counter’vs. prescription-strength nonsteroidal anti-inflammatory drugs in F rance. 2014;77(5):887-95.
13. Wilcox CM, Cryer B, Triadafilopoulos GJTJor. Patterns of use and public perception of over-the-counter pain relievers: focus on nonsteroidal antiinflammatory drugs. 2005;32(11):2218-24.

For peer review only

14. Castellsague J, Riera-Guardia N, Calingaert B, Varas-Lorenzo C, Fourrier-Reglat A, Nicotra F, et al. Individual NSAIDs and upper gastrointestinal complications. Drug safety. 2012;35(12):1127-46.
15. Vostinaru O. Adverse Effects and Drug Interactions of the Non-Steroidal Anti-Inflammatory Drugs. Nonsteroidal Anti-Inflammatory Drugs. 2017:17.
16. MacDonald T. Epidemiology and pharmacoeconomic implications of non-steroidal anti-inflammatory drug-associated gastrointestinal toxicity. Rheumatology. 2000;39(suppl_2):13-20.
17. Kish L. Sampling organizations and groups of unequal sizes. American sociological review. 1965:564-72.
18. Hamid S, Yakoob J, Jafri W, Islam S, Abid S, Islam MJJoPMA. Frequency of NSAID induced peptic ulcer disease. 2006;56(5):218.
19. Chatterjee S, Dureja GP, Kadhe G, Mane A, Phansalkar AA, Sawant S, et al. Cross-sectional study for prevalence of non-steroidal anti-inflammatory drug-induced gastrointestinal, cardiac and renal complications in India: interim report. 2015;8(3-4):216.
20. Tsujimoto S, Mokuda S, Matoba K, Yamada A, Jouyama K, Murata Y, et al. The prevalence of endoscopic gastric mucosal damage in patients with rheumatoid arthritis. 2018;13(7):e0200023.
21. Feng X, Wang X. Comparison of the efficacy and safety of non-steroidal anti-inflammatory drugs for patients with primary dysmenorrhea: A network meta-analysis. Molecular Pain. 2018;14:1744806918770320.
22. Dubois R, Melmed G, Henning J, Laine LJAp, therapeutics. Guidelines for the appropriate use of non-steroidal anti-inflammatory drugs, cyclo-oxygenase-2-specific inhibitors and proton pump inhibitors in patients requiring chronic anti-inflammatory therapy. 2004;19(2):197-208.
23. Ameade EPK, Amalba A, Mohammed BS. Prevalence of dysmenorrhea among University students in Northern Ghana; its impact and management strategies. BMC women's health. 2018;18(1):39.
24. Samba Conney C, Akwo Kretchy I, Asiedu-Danso M, Allotey-Babington GLJO, International G. Complementary and alternative medicine use for primary dysmenorrhea among senior high school students in the western region of Ghana. 2019;2019.
25. Moore NJIjocpS. Forty years of ibuprofen use. 2003(135):28-31.
26. Goldstein JL, Cryer BJD, healthcare, safety p. Gastrointestinal injury associated with NSAID use: a case study and review of risk factors and preventative strategies. 2015;7:31.
27. Jankovic SM, Aleksic J, Rakovic S, Aleksic A, Stevanovic I, Stefanovic-Stoimenov N, et al. Nonsteroidal antiinflammatory drugs and risk of gastrointestinal bleeding among patients on hemodialysis. 2009;22(4):502.
28. García Rodríguez LA, Hernández-Díaz SJAR, Therapy. The risk of upper gastrointestinal complications associated with nonsteroidal anti-inflammatory drugs, glucocorticoids, acetaminophen, and combinations of these agents. 2001;3(2):1-4.
29. Shorr RI, Ray WA, Daugherty JR, Griffin MRJAoim. Concurrent use of nonsteroidal anti-inflammatory drugs and oral anticoagulants places elderly persons at high risk for hemorrhagic peptic ulcer disease. 1993;153(14):1665-70.
30. Chang LJAp, therapeutics. Epidemiology and quality of life in functional gastrointestinal disorders. 2004;20:31-9.

For peer review only

31. Guo C-G, Leung WKJG, liver. Potential strategies in the prevention of nonsteroidal anti-inflammatory drugs-associated adverse effects in the lower gastrointestinal tract. 2020;14(2):179.

32. García-Rayado G, Navarro M, Lanas AJErocp. NSAID induced gastrointestinal damage and designing GI-sparing NSAIDs. 2018;11(10):1031-43.

For peer review only

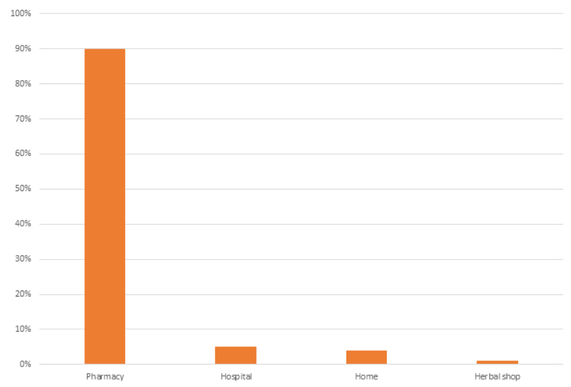

338x190mm (96 x 96 DPI)

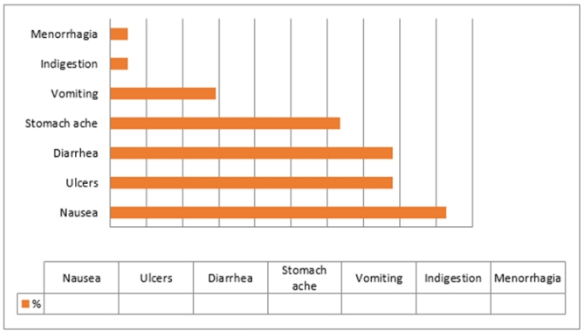

338x190mm (96 x 96 DPI)

STROBE Statement—Checklist of items that should be included in reports of ***cross-sectional studies***

	Item No	Recommendation
**Title and abstract**	1	(*a*) Indicate the study's design with a commonly used term in the title or the abstract-Page 1
		(*b*) Provide in the abstract an informative and balanced summary of what was done and what was found-Page 2
**Introduction**		
Background/rationale	2	Explain the scientific background and rationale for the investigation being reported-Page 3, 4
Objectives	3	State specific objectives, including any prespecified hypotheses-Page 4
**Methods**		
Study design	4	Present key elements of study design early in the paper-Page 4
Setting	5	Describe the setting, locations, and relevant dates, including periods of recruitment, exposure, follow-up, and data collection-Page 4,5
Participants	6	(*a*) Give the eligibility criteria, and the sources and methods of selection of participants-Page 5
Variables	7	Clearly define all outcomes, exposures, predictors, potential confounders, and effect modifiers. Give diagnostic criteria, if applicable-Page 6
Data sources/ measurement	8*	For each variable of interest, give sources of data and details of methods of assessment (measurement). Describe comparability of assessment methods if there is more than one group-Page 5
Bias	9	Describe any efforts to address potential sources of bias-Page 5
Study size	10	Explain how the study size was arrived at- page 5
Quantitative variables	11	Explain how quantitative variables were handled in the analyses. If applicable, describe which groupings were chosen and why-Page 6
Statistical methods	12	(*a*) Describe all statistical methods, including those used to control for confounding-Page 7
		(*b*) Describe any methods used to examine subgroups and interactions-Page 7
		(*c*) Explain how missing data were addressed-Page 7
		(*d*) If applicable, describe analytical methods taking account of sampling strategy-Page 7
		(*e*) Describe any sensitivity analyses-Page 7
**Results**		
Participants	13*	(a) Report numbers of individuals at each stage of study—eg numbers potentially eligible, examined for eligibility, confirmed eligible, included in the study, completing follow-up, and analysed-Page 7
Descriptive data	14*	(a) Give characteristics of study participants (eg demographic, clinical, social) and information on exposures and potential confounders-Page 7,8
		(b) Indicate number of participants with missing data for each variable of interest-Page 7
Outcome data	15*	Report numbers of outcome events or summary measures-Page 9
Main results	16	(*a*) Give unadjusted estimates and, if applicable, confounder-adjusted estimates and their precision (eg, 95% confidence interval). Make clear which confounders were adjusted for and why they were included-Page 12, 13

For peer review only - http://bmjopen.bmj.com/site/about/guidelines.xhtml

Other analyses	17	Report other analyses done—eg analyses of subgroups and interactions, and sensitivity analyses-Page 13
**Discussion**		
Key results	18	Summarise key results with reference to study objectives-page 14
Limitations	19	Discuss limitations of the study, taking into account sources of potential bias or imprecision. Discuss both direction and magnitude of any potential bias-Page 15
Interpretation	20	Give a cautious overall interpretation of results considering objectives, limitations, multiplicity of analyses, results from similar studies, and other relevant evidence-Page 14, 15
Generalisability	21	Discuss the generalisability (external validity) of the study results-Page 14, 15
**Other information**		
Funding	22	Give the source of funding and the role of the funders for the present study and, if applicable, for the original study on which the present article is based-Page 16

For peer review only

For peer review only - http://bmjopen.bmj.com/site/about/guidelines.xhtml

Self-reported gastrointestinal adverse effects of non-steroidal anti-inflammatory drugs in female students with dysmenorrhea at Makerere University: Prevalence, discontinuation and associated factors: A cross sectional study

Journal:	*BMJ Open*
Manuscript ID	bmjopen-2023-079660.R3
Article Type:	Original research
Date Submitted by the Author:	04-May-2024
Complete List of Authors:	Gobba, Solomon; Makerere University<br>Kibone, Winnie; Makerere University<br>Kiguba, Ronald; Makerere University College of Health Sciences, Pharmacology and Therapeutics
**Primary Subject Heading**:	Pharmacology and therapeutics
Secondary Subject Heading:	Reproductive medicine
Keywords:	Dysmenorrhea, Adverse events < THERAPEUTICS, REPRODUCTIVE MEDICINE

SCHOLARONE™
Manuscripts

I, the Submitting Author has the right to grant and does grant on behalf of all authors of the Work (as defined in the below author licence), an exclusive licence and/or a non-exclusive licence for contributions from authors who are: i) UK Crown employees; ii) where BMJ has agreed a CC-BY licence shall apply, and/or iii) in accordance with the terms applicable for US Federal Government officers or employees acting as part of their official duties; on a worldwide, perpetual, irrevocable, royalty-free basis to BMJ Publishing Group Ltd ("BMJ") its licensees and where the relevant Journal is co-owned by BMJ to the co-owners of the Journal, to publish the Work in this journal and any other BMJ products and to exploit all rights, as set out in our licence.

The Submitting Author accepts and understands that any supply made under these terms is made by BMJ to the Submitting Author unless you are acting as an employee on behalf of your employer or a postgraduate student of an affiliated institution which is paying any applicable article publishing charge ("APC") for Open Access articles. Where the Submitting Author wishes to make the Work available on an Open Access basis (and intends to pay the relevant APC), the terms of reuse of such Open Access shall be governed by a Creative Commons licence – details of these licences and which Creative Commons licence will apply to this Work are set out in our licence referred to above.

Other than as permitted in any relevant BMJ Author's Self Archiving Policies, I confirm this Work has not been accepted for publication elsewhere, is not being considered for publication elsewhere and does not duplicate material already published. I confirm all authors consent to publication of this Work and authorise the granting of this licence.

For peer review only

Self-reported gastrointestinal adverse effects of non-steroidal anti-inflammatory drugs in female students with dysmenorrhea at Makerere University: Prevalence, discontinuation and associated factors: A cross-sectional study

Solomon Gobba[1], Winnie Kibone[2], Ronald Kiguba[3]

Affiliations

[1] School of Health Sciences, College of Health Sciences, Makerere University, Kampala, Uganda

[2] School of Medicine, College of Health Sciences, Makerere University, Kampala, Uganda

[3] School of Biomedical Sciences, College of Health Sciences, Makerere University, Kampala, Uganda

***Corresponding author**

Gobba Solomon
School of Health Sciences, College of Health Sciences, Makerere University, Kampala, Uganda
Email: solomongobba@gmail.com

Author details

Solomon Gobba: solomongobba@gmail.com
Winnie Kibone: kibonewinnie@gmail.com
Ronald Kiguba: kiguba@gmail.com

For peer review only

Abstract

Background: Primary dysmenorrhea occurs in up to 50% of menstruating females. Non-Steroidal Anti-inflammatory Drugs (NSAIDS) are the most commonly used therapeutic remedies for dysmenorrhea in Uganda. However, NSAIDS are associated with a 3-to-5-fold increase in the risk of gastrointestinal (GI) adverse drug effects.

Objectives: We aimed to determine the prevalence and associated factors of self-reported NSAID-related GI adverse effects in female students who use NSAIDs in managing dysmenorrhea-associated pain at Makerere University.

Design: A Cross-sectional Study

Setting: Makerere University's main campus, situated North of Kampala, Uganda

Participants: 314 female students pursuing an undergraduate program at Makerere University and residing in different halls of residence and hostels.

Outcomes: Social demographic data, menstrual history, and treatment data. „.

Results: Overall, 314 valid responses were received from female students with a median age of 22 years (IQR: 18 – 29 years). The median age at menarche was 13 years (IQR: 9 – 18 years). 41% (n=129/314) of the respondents had used medication for dysmenorrhea, and 32% (n=41/129) of whom reported an NSAID-associated GI adverse effects with nausea being the most frequently reported (44%, n=18/41)

Factors independently associated with GI adverse effects were: age at menarche (p=0.026), duration of menstruation (P=0.030) and use of ibuprofen (p=0.005). Females taking ibuprofen for dysmenorrhoea were about 4 times as likely to have NSAID-associated GI adverse effects (AOR: 3.87, 95% CI: 1.51 – 9.91) than those who didn’t receive ibuprofen. Logistic regression was used to determine factors associated with self-reported adverse effects of NSAIDs among the female students. $p < 0.05$ was considered statistically significant.

For peer review only

Conclusion: We found a considerably high prevalence of NSAID-related GI adverse effects driven by factors such as age at menarche and ibuprofen use.

Keywords: NSAIDs, gastrointestinal (GI), adverse effect, dysmenorrhea, Uganda.

Strengths and Limitations of the study

- The study focuses on a public health issue (NSAID-related GI effects) in a specific population (female students with dysmenorrhea) in Uganda, where this information is limited.
- The study has clear objectives, elaborate study design and a well-defined methodology to determine the prevalence and associated factors of NSAID-related GI effects.
- The online survey method excludes students without internet access, potentially leading to a biased sample that may not represent the entire student population.
- Relying on self-reported data on medication use and GI effects can introduce some bias due to potential recall errors or misinterpretations

Introduction

Dysmenorrhea is defined as painful menstrual cramps and is classified into primary dysmenorrhea without any evident pathology and secondary dysmenorrhea with a pathology (1, 2). Pinpointing the exact prevalence remains elusive, as studies conducted in diverse populations show varying rates, spanning from 20% to 94% (3) Primary dysmenorrhea occurs in up to 50% of menstruating females and causes significant disruption in quality of life and absenteeism from school and workplaces affecting their productivity (4). The pain is attributed to uterine prostaglandins released by endometrial cells during uterine wall sloughing before the onset of menstruation (5). On account of the high prevalence of dysmenorrhea with a global prevalence ranging between 20% to 90%, dysmenorrhea is a serious public health burden (6). Studies have documented an equally higher

For peer review only

prevalence of dysmenorrhea estimated at 65.4%, 72.7% and 83.6% in Egypt, Ghana and Turkey respectively (7-9). In Uganda, the prevalence of dysmenorrhea has been estimated at 75.8% (10). Non-steroidal anti-inflammatory drugs (NSAIDs) remain the most commonly used therapeutic remedies for dysmenorrhea in Uganda because of their affordability, convenience of use and efficacy (11). NSAIDs are commonly used as over-the-counter drugs and may often not be prescribed (12, 13). NSAIDs relieve symptoms in up to 70% of women when used correctly. Furthermore, NSAIDs are the recommended first-line treatment in management of dysmenorrhea unless there are contraindications such as history of hypersensitivity to aspirin or other NSAIDs, serious comorbidity, and GI ulcers or bleeding (5).

The utilization of NSAIDs has been linked to a significant 3- to 5-fold increase in the risk of GI adverse effects (14). Dyspepsia with pyrosis, abdominal pain, nausea, anorexia, gastric erosions, ulcers, perforation, GI hemorrhage which may result in anemia are some of the commonly observed GI adverse effects in NSAID therapy (15). Studies have explored GI complications of NSAID-related treatment in cases of rheumatoid arthritis and osteoarthritis in the elderly (16) but little is known in relation to GI adverse drug effects related to NSAIDs therapy in the management of dysmenorrhea. Furthermore, university students practice more self-medication using NSAIDs for dysmenorrhea. In fact, recent publication showed a high prevalence of self-medication estimated at 37% commonly mefenamic acid (58%).

This study, aimed to determine the commonly used NSAIDs, the prevalence of self-reported NSAID-related GI adverse effects and the factors associated with self-reported NSAID-related GI adverse effects during the management of dysmenorrhea among female students at Makerere University, Kampala, Uganda.

Methods

For peer review only

Study design

A descriptive cross-sectional study was conducted, between 14th October 2020 and 29th October 2020 to determine the prevalence and factors associated with GI adverse effects to NSAIDs usage in the management of dysmenorrhea among female students at Makerere University.

Study setting

The study was conducted at Makerere University's main campus, situated North of Kampala, the capital city of Uganda, at the coordinates 0.3293° N, 32.5711° E. Makerere University is the largest public university in Uganda, attracting students from various regions of the country. The university accommodates over 40,000 students, both resident and non-resident. The main campus features twelve Halls of Residence, with six for male students and three for female students. Medical students are accommodated at the Mulago Hospital Complex. Approximately 5,000 resident undergraduates and 100 graduate students, accounting for 13% of the university's registered students, reside in the Halls of Residence. The remaining students either live in private hostels or commute from their homes. The age range of the majority of students falls between 20 and 30 years, encompassing a diverse representation of socio-economic statuses.

Study population

Female students pursuing an undergraduate program at Makerere University and residing in different halls of residence and hostels. The sample size of 314 participants was calculated using the modified Kish and Leslie formula(17) for finite population size, with an estimated prevalence of dysmenorrhea among university students (75.8%) , and a 10% adjustment was made to cater for nonresponse.

Selection criteria

For peer review only

All registered female students of Makerere University aged 18 years and older were eligible for the study after informed consent. Purposive sampling technique was used to select students into the study. Female students with primary or secondary amenorrhea, in menopause, with mental incapacitation or without access to internet were excluded from the study.

Data Collection

We used a self-administered questionnaire to collect data for this study. The questionnaire was pre-tested for comprehension and appropriateness among 15 students and adjusted accordingly to ensure quality data collection. The data were collected online via a Google survey document where participants received the survey information via a link shared with them in their WhatsApp groups and in their personal email inboxes. Eligible participants consented to the study and completed the survey. The submitted survey responses were automatically stored in an online data bank, accessible only to the Principal Investigator (PI) and the Supervisor. Additionally, the data were backed up on the Principal Investigator's Google Drive to ensure data security. The questionnaire variables were constituted in three parts: sociodemographic information, menstrual history, and treatment variables. These sections aimed to gather comprehensive data relevant to the study objectives.

Study Variables

Independent variables: social demographics (age, and history of pregnancy), menstrual history (dysmenorrhea, menarche, regularity and duration of menstrual periods) and treatment (painkillers used and their source, side effects).

For peer review only

Dependent variables: NSAID-related GI adverse effects.

Data quality control

To ensure that high quality data were captured from the volunteers, the data collection tool was pretested and adjusted to ensure convenience and ease of manoeuvre. Checks were incorporated within the document to ensure all questions were responded to and correctly answered to reduce the margins of error.

Data management

Data collection tools were checked for completeness and instantly submitted to a central data bank that tallied all responses received from the participants. The central databank was synchronized and backed up on the PI's google drive for secondary storage. Upon completion of the survey, the data were translated into Excel for data cleaning and initial analysis. The data were then exported to STATA Software (StataCorp LLC, USA) for further analysis.

Data analysis

All analyses were performed using Microsoft Excel 2016 and STATA Software. Numerical data were summarized as means and standard deviations if normally distributed; otherwise, median and interquartile ranges were used for non-normally distributed data. Categorical data were summarized as frequencies and proportions with their 95% confidence intervals. Associations between independent variables and dependent variables were assessed using the chi-square test and multivariable analysis using STATA 15.1 software. A P-Value of less than 0.05 was considered statistically significant.

For peer review only

Ethical and Regulatory Approval

We obtained ethical approval from of the School of Biomedical Sciences Research and Ethics Committee, Makerere University prior to study implementation, REC Approval Number SBS-REC -888. Participation in the study was voluntary and participants provided informed consent prior to enrolment into the study. All information was kept confidential and used for research purposes only.

Patient and Public Involvement

Patients and the public were at the centre of this research, our research questions and outcome measures were shaped by findings from previous studies which often reflect patient-reported outcomes, adverse events, and medication-related errors. We aimed to develop research objectives that were meaningful, relevant, and actionable in addressing medication safety issues from a patient-centered standpoint.

We conducted a questionnaire pretest session with a select group of participants to refine study questionnaire which ensured that the study design was sensitive to patient preferences and experiences.

The results of the study will be disseminated to study participants and the general public through various channels to ensure accessibility and comprehension. This will include the distribution of lay summaries written in plain language, presentations at patient-focused conferences, and publication of study findings in open-access journals.

For peer review only

193 **Results**

194 **Characteristics of Participants**

195 Overall, a total of 314 participants responded to the study. The median age was 22 years (IQR: 21-
196 23). The majority came from nuclear families (62%, 194/314). Only 8% (26/314) of the
197 participants had a history of pregnancy. The median age at menarche was 13 years (IQR: 12-14)
198 with range of 9 to 18 years and more than half (55%, 173/314) experienced regular periods every
199 time. Nearly all female students were aware of dysmenorrhoea (99%, 310/314). **Table 1**
200 summarizes the baseline characteristics of participants.

201 **Table 1. Demographic and Menstrual Characteristics of Participants**

**Variable**	**Frequency (n)**	**Percentage (%)**
Age of respondents, years (Median, IQR)	22	21-23
18-21	70	22.3
22-25	168	53.5
26-29	40	12.7
30 and above	36	11.5
Age at menarche, years (median, IQR)	13	12-14
9-11	24	7.6
12-14	206	65.6
15 and above	84	26.8
Type of family		
Nuclear	194	61.8
Extended	81	25.8
Single parent	37	11.8
Divorced parents	2	0.6
History of pregnancy		
Yes	26	8.3
No	288	91.7
Awareness on dysmenorrhoea		
Yes	310	98.7
No	4	1.3
Regular menstrual periods		
Every time	173	55.1
Most times	91	29.0
Sometimes	34	10.8
Rarely	16	5.1

For peer review only

Duration of menstrual periods (days)		
1-2	10	3.2
3-4	95	30.3
5-6	76	24.2
7-8	19	6.1
9 and above	1	0.3

Patterns of dysmenorrhea and medication use

The majority of participants (42%, 132/314) reported experiencing menstrual cramps every time and about a quarter (23%, 71/314) reported using medicines to reduce pain during menstrual pain every time. Most (87%, 273/314) of the participants reported experiencing menstrual cramps in the past three months of whom nearly half (47%, 129/273) reported using a medication for menstrual cramps with Ibuprofen and paracetamol being the most frequently used medications (40%, 52/129 and 40%, 52/129 respectively). The majority (90%, 116/129) bought the medication from pharmacies. **Figure 1** shows the sources of the medication. **Table 2** summarizes the patterns of dysmenorrhea and medication use among female students at Makerere University.

Table 2. Patterns of dysmenorrhoea and medication use among female students at Makerere University

**Variable**	**Frequency**	**%**
Experienced menstrual pains/ cramps/ dysmenorrhea		
Every time	132	42.0
Most times	71	22.6
Sometimes	76	24.2
Rarely	35	11.2
Frequency of use of medicines (pain killers) to reduce pain during your menstrual period		
Every time	71	22.6
Most times	52	16.6
Sometimes	60	19.1
Rarely	131	41.7
Experienced menstrual cramps in the past 3 months		

For peer review only

Yes	273	86.9
No	41	13.1
How long ago the most recent period was (days)		
1-5	180	57.32
6-10	98	31.21
11 and above	36	11.47
Use of medication		
Yes	129	47.3
No	144	52.8
Medicines used for menstrual pains/cramps/ dysmenorrhea		
Ibuprofen	52	40.3
Paracetamol	52	40.3
Diclofenac	38	29.5
Piroxicam	26	20.2
Mefenamic Acid	9	7.0
Aspirin	6	4.7
Indomethacin	3	2.3
Dynapar	3	2.3
Brustan	3	2.3
Tramadol	2	1.6
Herb	1	0.8
Meloxicam	1	0.8

Prevalence of NSAID-Related GI adverse effects

Data of 129 female students who had used a medication while having dysmenorrhoea in the past three months were analysed. The prevalence of NSAID-associated GI adverse effects was 32% (n=41/129). Nausea (44%, n=18/41), ulcers (39%, n=16/41) and diarrhoea (39%, n=16/41) were the most frequently reported adverse, effects. **Figure 2**. Ibuprofen (59%, n=24/41), diclofenac (46%, n=19/41), paracetamol (15%, n=6/41), indomethacin (7%, n=3/41) and aspirin (2%, n=1/41) were implicated for the adverse effects.

For peer review only

Factors associated with NSAID-associated gastrointestinal adverse effects

At multivariable logistic regression, **Table 3**. Factors independently associated with GI adverse effects were: age at menarche (AOR: 1.57, 95% CI: 1.05 - 2.34), duration of menstruation (AOR: 1.57, 95% CI: 1.05 - 2.36). and use of ibuprofen (AOR: 3.87, 95% CI: 1.51 - 9.91). Females taking ibuprofen for dysmenorrhoea were about 4 times more likely to have NSAID-associated GIT adverse effects (AOR: 3.87, 95% CI: 1.51 – 9.91).

Table 3. Multivariable logistic regression showing factors associated with NSAID-associated GI adverse effects among female students at Makerere University

**Variable**	**AOR**	**95% CI**	**P**
Current age	1.00	0.74 - 1.34	0.994
Age at menarche	1.57	1.05 - 2.34	0.026
Duration of menstruation	1.57	1.05 - 2.36	0.030
Regular menstrual periods			
Every time	1.00		
Most times	1.33	0.49 - 3.59	0.574
Sometimes	0.28	0.03 - 2.76	0.275
Rarely	5.87	0.37 - 93.35	0.210
Use of NSAID for pain relief			
Every time	1.00		
Most times	0.69	0.25 - 1.90	0.470
Sometimes	0.37	0.09 - 1.61	0.187
Ibuprofen	3.87	1.51 - 9.91	0.005

Note: AOR- adjusted odds ratio, CI- confidence interval, NSAID- non steroidal anti-inflammatory disease

For peer review only

Discontinuation for NSAID-associated GI adverse effects

Overall, 68% (88/314) of the female students reported having been advised to discontinue NSAIDs (**Table 4**). Ulcers were the most common reason for discontinuation in 29 students (33%, 29/88). Of concern, some (8%, 7/88) of the students claimed that NSAIDs could cause infertility.

Table 4. Discontinuation of NSAID usage among female students at Makerere University

Variable	Frequency	%
Received advice to stop using a medication because of possible GI adverse effect		
Yes	88	68.0
No	41	32.0
Reason for advice to stop use of the NSAID?		
Ulcers	29	33.0
Infertility	7	8.0
Tolerance	3	3.4
Not good for women	2	2.3
Allergy	1	1.1
Can cause fibroids	1	1.1
Can cause cancer	1	1.1
Headache	1	1.1
Nausea	1	1.1
Menorrhagia	1	1.1
Vomiting	1	1.1
Nephrotic syndrome	1	1.1

Discussion

This study, aimed to determine the prevalence and factors associated with self-reported non-steroidal anti-inflammatory drugs (NSAIDs)-related gastrointestinal (GI) adverse effects among female students with dysmenorrhea-associated pain at Makerere University. We found a high prevalence of GI adverse effects, reaching 32%. The factors significantly associated with adverse effects included age at menarche, duration of menstruation, and ibuprofen use for pain relief.

For peer review only

Whereas there is a paucity of data on NSAID relatedGI adverse effects among female university students, previous studies conducted in India, Pakistan and Japan among patients using NSAIDs reported a prevalence of GI adverse effects, such as peptic and gastric ulcers, estimated at 30.08%, 14.7% and 16.7% respectively (18-20). In contrast to our findings, previous studies have showed that diclofenac was the commonest NSAID associated with GI adverse effects (18). The difference could be attributed to variations in the most commonly used NSAID among participants in different studies and variations in study designs. A network meta-analysis by X. Feng and X. Wang revealed that Tiaprofenic acid and mefenamic acid were indicated as the safest NSAIDs drugs, whereas indomethacin was the least safe with a higher likehood to cause mild gastrointestinal discomfort (21). In addition, a significant increase of about 2.5-5.0% in the occurrence of GI adverse effects has been observed in individuals with a history of GI incidents (22).

Comparable to findings from Ghana where the prevalence of medication use for dysmenorrhea was 46%, our study observed that 47% (129/273) of participants used medications (23). However, the frequency of the use of medication to alleviate pain greatly varied amongst the participants and could be attributed to the intensity of pain and unregulated drug use as indicated by our finding that about 90% of participants acquired the painkillers over the counter at the pharmacy (24). Contrary to a study by Moore and colleagues that showed that ibuprofen was a much safer drug than paracetamol, our study showed that females taking ibuprofen for dysmenorrhoea were about 4 times more likely to have NSAID-associated GIT adverse effects (59%) than paracetamol (15%) (25).

Whereas our study deduced that 1-year increase in the age at menarche and one-day increase in the duration of menstruation were 1.57 more likely associated with having NSAID-associated GIT adverse effects, other factors implicated in other studies include; *Helicobacter pylori* infection,

For peer review only

274 high dose or multiple NSAID use, history of upper GI injury, receiving hemodialysis and
275 anticoagulant, oral corticosteroid or selective serotonin reuptake inhibitor use (26-29).

276 GI adverse effects are associated with increased physician visits, risk of GI surgery, high health
277 care costs and poorer health related quality of life (HRQoL) (30). However, potential strategies
278 such as the use of GI sparing NSAIDs, use of muco-protective drugs and alternative medications
279 or no pharmacological remedies for pain relief have been adopted in order to prevent NSAID
280 induced GI adverse effects (31, 32).

281 Our study contributes to the existing literature on the current status of NSAID-related GI effects
282 among female university students in Uganda. However, there were some limitations. Due to the
283 COVID-19 pandemic, the study was conducted online. Therefore, a limited number of students
284 with internet access participated. This also limited the nature of study participant selection as we
285 were unable to use probability sampling methods, the sampling technique used may not have given
286 us the best representative study population. In addition, the exclusive use of a quantitative design
287 in our study prevented us from exploring important perspectives that could have been derived from
288 a qualitative study. While we performed statistical analyses to examine variable associations, we
289 did not explicitly test for potential confounding and interaction effects, which may exist and have
290 the potential to influence our findings.

291 **Conclusion**

292 In this study, we found a considerably high prevalence of NSAID-related GI adverse effects
293 associated with; age at menarche, longer duration of menstruation, and ibuprofen use among
294 female students at Makerere University experienced dysmenorrhea. Therefore, efforts directed
295 towards mass education of females about the prevention, early identification and management of

For peer review only

NSAID-related GI adverse effects are necessary. We recommend further exploration of alternative strategies for pain relief for females with dysmenorrhea.

Acknowledgments

The authors acknowledge the contribution of the following team of individuals in the data collection process. The team is as follows; Dr. Haruna Kiryowa, Mr. David Bakka, Mr. Gerald Akena, Mr. Samuel Ssenkungu, Ms. Catherine Aiya Lalam and Ms. Gillian Sheila Amuge,

Contributorship statement

All authors contributed significantly to study conceptualisation, data collection, analysis, interpretation, and manuscript writing.

GS, KW and RK contributed to the study concept and design, and developed the questionnaire. GS and KW recruited collaborators for survey distribution and data collection. GS, KR and KW contributed to data acquisition, analysis and interpretation. KW wrote the initial draft and all authors participated in subsequent writing and critical revision of the manuscript.

RK oversaw the project implementation. KW managed project administration.

All authors approved the final version to be published and are accountable for all aspects of the work.

Competing interests

All authors declare that they have no conflict of interest.

Funding

This research was financially supported by Medical Research Council (MR/V03510X/1).

For peer review only

Data sharing statement

No additional data are available.

Figure Captions

Figure 1. Sources of medication for menstrual cramps relief among female students at Makerere University

Figure 2. Frequency of NSAID-associated GI adverse effects among female students at Makerere University

References

1. Kumar KH, Elavarasi PJJoAC, Insights R. Definition of pain and classification of pain disorders. 2016;3(3):87-90.
2. Smith RP. The clinical classification and causes of dysmenorrhea. Dysmenorrhea and Menorrhagia: Springer; 2018. p. 55-64.
3. Parry K, Armour M, Smith CA, MacMillan F, Manohar N, Holmes K, et al. The Prevalence and Academic Impact of Dysmenorrhea in 21,573 Young Women: A Systematic Review and Meta-Analysis. Journal of Women's Health (15409996). 2019;28(8).
4. Dawood MY. Primary dysmenorrhea: advances in pathogenesis and management. Obstetrics & Gynecology. 2006;108(2):428-41.
5. Proctor MML, Farquhar CM. Dysmenorrhoea. BMJ clinical evidence. 2007;2007.
6. Vincenzo De Sanctis M, Soliman A, Bernasconi S, Bianchin L, Bona G, Bozzola M, et al. Primary dysmenorrhea in adolescents: prevalence, impact and recent knowledge. 2015;13(2):465-73.
7. Unsal A, Ayranci U, Tozun M, Arslan G, Calik EJUjoms. Prevalence of dysmenorrhea and its effect on quality of life among a group of female university students. 2010;115(2):138-45.
8. Ameade EPK, Amalba A, Mohammed BSJBWsH. Prevalence of dysmenorrhea among University students in Northern Ghana; its impact and management strategies. 2018;18(1):1-9.
9. Nooh AMJMEFSJ. Menstrual disorders among Zagazig university students, Zagazig, Egypt. 2015;20(3):198-203.
10. Nakame RM, Kiwanuka F, Robert AJNo. Dysmenorrhoea among students aged 18–45 years attending University in Uganda: A cross-sectional multicenter study of three Universities in Uganda. 2019;6(2):268-75.
11. Alshakka MA, Badullah WF, Alolayan SO, Mahmoud MAJBR. Prescribing patterns of non-steroidal anti-inflammatory drugs (NSAIDs) at outpatient departments of four hospitals. 2018;29(19):3643-7.

For peer review only

12. Duong M, Salvo F, Pariente A, Abouelfath A, Lassalle R, Droz C, et al. Usage patterns of ‘over-the-counter’vs. prescription-strength nonsteroidal anti-inflammatory drugs in F rance. 2014;77(5):887-95.

13. Wilcox CM, Cryer B, Triadafilopoulos GJTJor. Patterns of use and public perception of over-the-counter pain relievers: focus on nonsteroidal antiinflammatory drugs. 2005;32(11):2218-24.

14. Castellsague J, Riera-Guardia N, Calingaert B, Varas-Lorenzo C, Fourrier-Reglat A, Nicotra F, et al. Individual NSAIDs and upper gastrointestinal complications. Drug safety. 2012;35(12):1127-46.

15. Vostinaru O. Adverse Effects and Drug Interactions of the Non-Steroidal Anti-Inflammatory Drugs. Nonsteroidal Anti-Inflammatory Drugs. 2017:17.

16. MacDonald T. Epidemiology and pharmacoeconomic implications of non-steroidal anti-inflammatory drug-associated gastrointestinal toxicity. Rheumatology. 2000;39(suppl_2):13-20.

17. Kish L. Sampling organizations and groups of unequal sizes. American sociological review. 1965:564-72.

18. Hamid S, Yakoob J, Jafri W, Islam S, Abid S, Islam MJJoPMA. Frequency of NSAID induced peptic ulcer disease. 2006;56(5):218.

19. Chatterjee S, Dureja GP, Kadhe G, Mane A, Phansalkar AA, Sawant S, et al. Cross-sectional study for prevalence of non-steroidal anti-inflammatory drug-induced gastrointestinal, cardiac and renal complications in India: interim report. 2015;8(3-4):216.

20. Tsujimoto S, Mokuda S, Matoba K, Yamada A, Jouyama K, Murata Y, et al. The prevalence of endoscopic gastric mucosal damage in patients with rheumatoid arthritis. 2018;13(7):e0200023.

21. Feng X, Wang X. Comparison of the efficacy and safety of non-steroidal anti-inflammatory drugs for patients with primary dysmenorrhea: A network meta-analysis. Molecular Pain. 2018;14:1744806918770320.

22. Dubois R, Melmed G, Henning J, Laine LJAp, therapeutics. Guidelines for the appropriate use of non-steroidal anti-inflammatory drugs, cyclo-oxygenase-2-specific inhibitors and proton pump inhibitors in patients requiring chronic anti-inflammatory therapy. 2004;19(2):197-208.

23. Ameade EPK, Amalba A, Mohammed BS. Prevalence of dysmenorrhea among University students in Northern Ghana; its impact and management strategies. BMC women's health. 2018;18(1):39.

24. Samba Conney C, Akwo Kretchy I, Asiedu-Danso M, Allotey-Babington GLJO, International G. Complementary and alternative medicine use for primary dysmenorrhea among senior high school students in the western region of Ghana. 2019;2019.

25. Moore NJIjocpS. Forty years of ibuprofen use. 2003(135):28-31.

26. Goldstein JL, Cryer BJD, healthcare, safety p. Gastrointestinal injury associated with NSAID use: a case study and review of risk factors and preventative strategies. 2015;7:31.

27. Jankovic SM, Aleksic J, Rakovic S, Aleksic A, Stevanovic I, Stefanovic-Stoimenov N, et al. Nonsteroidal antiinflammatory drugs and risk of gastrointestinal bleeding among patients on hemodialysis. 2009;22(4):502.

28. García Rodríguez LA, Hernández-Díaz SJAR, Therapy. The risk of upper gastrointestinal complications associated with nonsteroidal anti-inflammatory drugs, glucocorticoids, acetaminophen, and combinations of these agents. 2001;3(2):1-4.

For peer review only

29. Shorr RI, Ray WA, Daugherty JR, Griffin MRJAoim. Concurrent use of nonsteroidal anti-inflammatory drugs and oral anticoagulants places elderly persons at high risk for hemorrhagic peptic ulcer disease. 1993;153(14):1665-70.

30. Chang LJAp, therapeutics. Epidemiology and quality of life in functional gastrointestinal disorders. 2004;20:31-9.

31. Guo C-G, Leung WKJG, liver. Potential strategies in the prevention of nonsteroidal anti-inflammatory drugs-associated adverse effects in the lower gastrointestinal tract. 2020;14(2):179.

32. García-Rayado G, Navarro M, Lanas AJErocp. NSAID induced gastrointestinal damage and designing GI-sparing NSAIDs. 2018;11(10):1031-43.

For peer review only

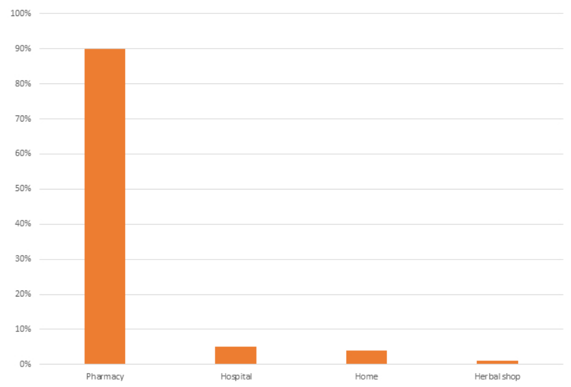

338x190mm (96 x 96 DPI)

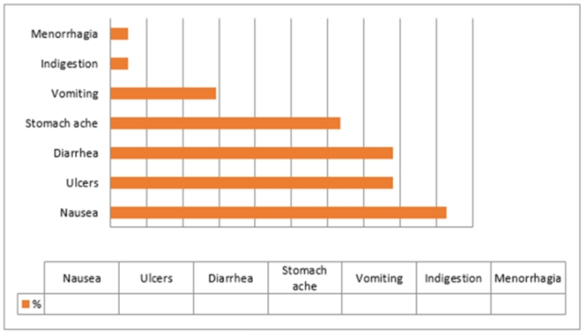

338x190mm (96 x 96 DPI)

STROBE Statement—Checklist of items that should be included in reports of ***cross-sectional studies***

	Item No	Recommendation
**Title and abstract**	1	(*a*) Indicate the study's design with a commonly used term in the title or the abstract-Page 1
		(*b*) Provide in the abstract an informative and balanced summary of what was done and what was found-Page 2
**Introduction**		
Background/rationale	2	Explain the scientific background and rationale for the investigation being reported-Page 3, 4
Objectives	3	State specific objectives, including any prespecified hypotheses-Page 4
**Methods**		
Study design	4	Present key elements of study design early in the paper-Page 4
Setting	5	Describe the setting, locations, and relevant dates, including periods of recruitment, exposure, follow-up, and data collection-Page 4,5
Participants	6	(*a*) Give the eligibility criteria, and the sources and methods of selection of participants-Page 5
Variables	7	Clearly define all outcomes, exposures, predictors, potential confounders, and effect modifiers. Give diagnostic criteria, if applicable-Page 6
Data sources/ measurement	8*	For each variable of interest, give sources of data and details of methods of assessment (measurement). Describe comparability of assessment methods if there is more than one group-Page 5
Bias	9	Describe any efforts to address potential sources of bias-Page 5
Study size	10	Explain how the study size was arrived at- page 5
Quantitative variables	11	Explain how quantitative variables were handled in the analyses. If applicable, describe which groupings were chosen and why-Page 6
Statistical methods	12	(*a*) Describe all statistical methods, including those used to control for confounding-Page 7
		(*b*) Describe any methods used to examine subgroups and interactions-Page 7
		(*c*) Explain how missing data were addressed-Page 7
		(*d*) If applicable, describe analytical methods taking account of sampling strategy-Page 7
		(*e*) Describe any sensitivity analyses-Page 7
**Results**		
Participants	13*	(a) Report numbers of individuals at each stage of study—eg numbers potentially eligible, examined for eligibility, confirmed eligible, included in the study, completing follow-up, and analysed-Page 7
Descriptive data	14*	(a) Give characteristics of study participants (eg demographic, clinical, social) and information on exposures and potential confounders-Page 7,8
		(b) Indicate number of participants with missing data for each variable of interest-Page 7
Outcome data	15*	Report numbers of outcome events or summary measures-Page 9
Main results	16	(*a*) Give unadjusted estimates and, if applicable, confounder-adjusted estimates and their precision (eg, 95% confidence interval). Make clear which confounders were adjusted for and why they were included-Page 12, 13

For peer review only - http://bmjopen.bmj.com/site/about/guidelines.xhtml

Other analyses	17	Report other analyses done—eg analyses of subgroups and interactions, and sensitivity analyses-Page 13
**Discussion**		
Key results	18	Summarise key results with reference to study objectives-page 14
Limitations	19	Discuss limitations of the study, taking into account sources of potential bias or imprecision. Discuss both direction and magnitude of any potential bias-Page 15
Interpretation	20	Give a cautious overall interpretation of results considering objectives, limitations, multiplicity of analyses, results from similar studies, and other relevant evidence-Page 14, 15
Generalisability	21	Discuss the generalisability (external validity) of the study results-Page 14, 15
**Other information**		
Funding	22	Give the source of funding and the role of the funders for the present study and, if applicable, for the original study on which the present article is based-Page 16

For peer review only

For peer review only - http://bmjopen.bmj.com/site/about/guidelines.xhtml
